# Extending the Basic Local Independence Model to Polytomous Data

**DOI:** 10.1007/s11336-020-09722-5

**Published:** 2020-09-21

**Authors:** Luca Stefanutti, Debora de Chiusole, Pasquale Anselmi, Andrea Spoto

**Affiliations:** 1 Department of Philosophy, Sociology, Pedagogy, and Applied Psychology, University of Padua, Padua, Italy; 2 Department of General Psychology, Via Venezia, 8, 35131 Padua, Italy

**Keywords:** polytomous knowledge space theory, basic local independence model, probabilistic structures, polytomous items, Likert scale, psychological assessment

## Abstract

A probabilistic framework for the polytomous extension of knowledge space theory (KST) is proposed. It consists in a probabilistic model, called *polytomous local independence model*, that is developed as a generalization of the basic local independence model. The algorithms for computing “maximum likelihood” (ML) and “minimum discrepancy” (MD) estimates of the model parameters have been derived and tested in a simulation study. Results show that the algorithms differ in their capability of recovering the true parameter values. The ML algorithm correctly recovers the true values, regardless of the manipulated variables. This is not totally true for the MD algorithm. Finally, the model has been applied to a real polytomous data set collected in the area of psychological assessment. Results show that it can be successfully applied in practice, paving the way to a number of applications of KST outside the area of knowledge and learning assessment.

**Electronic supplementary material:**

The online version of this article (10.1007/s11336-020-09722-5) contains supplementary material, which is available to authorized users.

## Introduction

Knowledge space theory (KST) was introduced in 1985 by Jean-Paul Doignon and Jean-Claude Falmagne (Doignon and Falmagne 1985, 1999; Falmagne and Doignon 2011) with the aim of building “an efficient machine for the assessment of knowledge” (Doignon and Falmagne 1999, Preface). This is pursued by developing a nonnumerical representation of the individual knowledge called *knowledge state* and defined as the set of all those problems that a student is capable of solving. Although the very first formulation of the theory was essentially deterministic, after few years the probabilistic concepts related to the concrete application of the theory were formalized (Falmagne and Doignon 1988a; b). Some probabilistic models have been developed, the most popular being the basic local independence model (BLIM; Falmagne and Doignon 1988a). The development of both the deterministic and the probabilistic frameworks leads KST to become a rigorous and effective tool for both the assessment of knowledge and the implementation of customized learning programs.

One of the core characteristics of KST is that it is applied to dichotomous problems. While this response format is well suited for the assessment of knowledge, it appears to be restrictive for the recent applications of the theory such as psychological assessment (e.g., Bottesi et al. 2015; Falmagne et al. Submitted; Spoto et al. 2010) and social sciences (Martin and Wiley 2000; Wiley and Martin 1999). In fact, the use of polytomous items is quite common in these fields. Therefore, the generalization of both the deterministic and probabilistic concepts of KST to the case of polytomous items could, indeed, pave the way to its application to data that are much more sophisticated than a mere dichotomy.

The generalization of the deterministic concepts of KST to the case of polytomous items has been already approached in the literature. Drawing upon a first and almost isolated attempt by Schrepp (1997) to formalize KST in a polytomous fashion, Stefanutti et al. (2020) proposed a new formulation of the polytomous KST (PolyKST). One element that immediately emerged from this extension, given the combinatorial nature of KST, is the chance to deal with ordinal measures without the assumption of either any kind of continuity in the measured latent trait (as it happens, for instance, in traditional item response theory), or any additive properties of item responses (as it happens, for instance, in classical test theory with Likert scale items). Both these issues shed further light on some debated issues of the classically made assumptions in psychometric measures, and they show that an approach based on the conceptual framework of KST could fruitfully account for the great amount of information provided by polytomous items.

This article is aimed at filling the last gap in the generalization of KST to the case of polytomous data by providing an extension of the BLIM to polytomous items.

KST is not the only framework providing a nonnumerical representation of individual knowledge. A prominent role is also played by cognitive diagnostic models (CDMs; Bolt 2007; de la Torre 2009b; DiBello and Stout 2007; Junker and Sijtsma 2001; Tatsuoka 1990), in which the knowledge of an individual is described as the set of attributes she has, rather than as the set of items she is able to solve. This theory was developed in the same years of KST and presents some overlapping with it, although there have been rare interactions between the two. Recently, Heller et al. (2015, 2016) have pointed out the connection between KST and CDMs highlighting that the two frameworks not only share the aim of a nonnumerical assessment of knowledge, but also some of the probabilistic models that were developed with this aim. More in detail, the competence-based local independence model (CBLIM; Heller et al. 2015) developed in KST is equivalent to the multiple strategy deterministic input, noisy AND gate (MS-DINA; de la Torre and Douglas 2008) model which has, as special cases, the deterministic input, noisy AND gate (DINA; Haertel 1989; Junker and Sijtsma 2001) model and the deterministic input noisy OR gate (DINO; Templin and Henson 2006) model. All of these models are well suited for dichotomous data.

The problem of extending the theory to the case of polytomous data has been approached also within CDM. In fact, some of the most recent CDM models are for polytomous data (see e.g., Chen and Zhou 2017; Chen and de la Torre 2018; de la Torre 2009a; DiBello et al. 1993; von Davier 2008). They differ from one another in the way they establish the association between attributes and response categories. Such attribution, in turn, depends on the type of items for which the models are meant (e.g., nominal or ordinal). In the spirit of the early KST, the probabilistic approach presented in this paper has a behavioral focus stating no assumptions on underlying attributes. In this respect, our proposal is different from any existing CDM model for polytomous items. Furthermore, it turns out to be general enough to be applied with various types of polytomous items (e.g., both categorical and ordinal polytomous items). Ma and de la Torre (2016) proposed a sequential CDM for polytomous items which can accommodate both ordinal and nominal responses. In that model, ordinal response categories are assumed to be attained sequentially, from the lowest to the highest. While this assumption is plausible for partial credit data, it may not be so for rating data. In our proposal, there is no assumption about the particular mechanism underlying ordinal responses. In addition, the sequential CDM deals with nominal responses by assuming that all attributes required by an item are needed by each response category of that item (i.e., exactly the same attributes are assigned to all response categories of an item). Thus, it is not clear to what extent the particular response to the item is informative about the attribute profile of an individual. Moreover, there seems to be no substantial difference between this type of polytomous nominal items and standard dichotomous ones, in the sense that there is no loss of information when collapsing polytomous nominal item categories into dichotomous ones. As already stated, in our proposal no assumptions are made about the attributes underlying the response categories.

The paper is organized as follows. Sections 2.1 and 2.2 introduce, respectively, the main deterministic and probabilistic issues of the dichotomous KST. Section 2.3 presents the main results obtained in the generalization of the theory to the polytomous case. Section 3 describes a proposal for the extension of the probabilistic concepts of dichotomous KST to the polytomous case, culminating in the *polytomous local independence model* (called PoLIM). Sections 4 and 5 present the results of an application of the PoLIM to simulated and real data, respectively. Finally, all the theoretical and practical results as well as a list of open issues are discussed in Sect. 6.

## Backgrounds

### Deterministic Concepts in KST

In KST (Doignon and Falmagne 1985, 1999; Falmagne and Doignon 2011), the *knowledge domain* *Q* is the set of items that can be formulated in order to explore students’ knowledge with respect to a certain topic. In the classical formulation of KST, the answers to items are dichotomously classified as correct or incorrect. The *knowledge state* of an individual is the set $K \subseteq Q$ of items she is able to solve. A *knowledge structure* is a pair $(Q, \mathcal {K})$ where *Q* is the knowledge domain and $\mathcal {K}$ is a collection of subsets of *Q*. The minimal structure on *Q* is the collection containing only $\emptyset$ and *Q*. The maximal structure is the power set $2^Q$ (i.e., the collection of all subsets of *Q*, including $\emptyset$ and *Q* itself). Within these two extreme cases, a structure can be defined by a precedence relation, named the *surmise relation*, among the items in *Q*, which provides the admissible knowledge states $K \in \mathcal {K}$. An item *p* is a prerequisite of another item *q* iff $(q\in K) \Rightarrow (p \in K)$ for all $K \in \mathcal {K}$.

An example could be useful to clarify the above introduced concepts. Let us consider the knowledge domain $Q_1=\{a, b, c, d, e\}$ containing five problems about a specific topic. The knowledge structure $\mathcal {K}_1$ defined on $Q_1$ contains the following states:

$$\begin{aligned} \mathcal {K}_1 = \{\emptyset , \{a\},\{b\},\{a,b\},\{a,d\},\{b,c\},\{b,d\},\{a,b,c\},\{a,b,d\},\{b,c,d\},\{a,b,c,d\},Q_1\}. \end{aligned}$$

Notice that both $\emptyset$ and $Q_1$ are states in $\mathcal {K}_1$. Moreover, out of the $2^{|Q_1|}=2^5=32$ different subsets of the power set on $Q_1$, only 12 belong to $\mathcal {K}_1$. This is due to the prerequisite relations defined among the items in $Q_1$. For instance, it can be observed that items *a* and *b* are prerequisites of items *d* and *c*, respectively. In fact, there is no state in $\mathcal {K}_1$ either containing *d* and not containing *a*, or containing *c* and not *b*. Moreover, all the items are prerequisites of item *e*, which is contained only in the state $Q_1$. In other words, to solve item *e* a student has to master all the remaining items. On the other hand, items *a* and *b* have no prerequisites, that is, it is possible to solve any of them and not to master any other item in $Q_1$.

Whenever a structure is closed under both union and intersection, it is a *quasi-ordinal knowledge space*. The structure $\mathcal {K}_1$ of the example introduced above is closed under both union and intersection: Any union of states in $\mathcal {K}_1$ produces a new state already contained in $\mathcal {K}_1$; moreover, any intersection of states in $\mathcal {K}_1$ produces a new state already contained in $\mathcal {K}_1$. Therefore, it is a quasi-ordinal knowledge space.

The theorem by Birkhoff (1937) established a one-to-one correspondence between the set of all the quasi-ordinal knowledge spaces defined on the domain *Q* and the set of all the surmise relations (i.e., quasi-order relations) on *Q*. Whenever a structure is closed only under set union, it is a *knowledge space*; whenever it is closed only under intersection, it is called a *closure space*. Doignon and Falmagne (1985) established a one-to-one correspondence between the set of all the knowledge spaces on *Q*, and all the surmise functions defined on *Q*. The crucial difference between surmise functions and surmise relations is that the latter admit only one set of prerequisites for each item $q \in Q$.

A knowledge structure $\mathcal {K}$ is said to be backward graded (BG) in an item *q* if $K \setminus \{q\} \in \mathcal {K}$ for every $K \in \mathcal {K}$ (Spoto et al. 2012; 2013). Thus, if the item *q* is removed from any state in $\mathcal {K}$, then the result will still be a state in $\mathcal {K}$. Dually, a structure $\mathcal {K}$ is forward graded (FG) in an item *q* if $K \cup \{q\} \in \mathcal {K}$ for every $K \in \mathcal {K}$. Thus, if the item *q* is added to any state in $\mathcal {K}$, the resulting subset of items will be a state in $\mathcal {K}$. Forward and backward gradedness describes a quite frequent condition in knowledge structures. For instance, quasi-ordinal spaces are both FG and BG in at least one item, whereas knowledge spaces containing singletons are FG in the items contained in such singletons. As described before, the structure $\mathcal {K}_1$ in the previous example is a quasi-ordinal knowledge space. It can be observed that $\mathcal {K}_1$ is FG in both items *a* and *b*, and it is BG in item *e*. In fact by adding, for instance, item *a* to any state not containing it, the result is a state already contained in $\mathcal {K}_1$; if we remove item *e* from the only state containing it, that is, the state $Q_1$, we obtain $\{a,b,c,d\}$ which belongs to $\mathcal {K}_1$. The same does not hold for any other item in $Q_1$. The BLIM, which is the mostly used probabilistic model in KST, has been found to be not identifiable for FG or BG knowledge structures (Heller 2017; Spoto et al. 2012, 2013; Stefanutti et al. 2018).

### The Basic Local Independence Model

Let $\mathcal {K}$ be the knowledge structure defined on the domain *Q*. Considering a certain population of students, it is plausible to assume the existence of a probability distribution $\pi$ (i.e., $\pi _K \ge 0$ for all $K \in \mathcal {K}$ and $\sum _{K \in \mathcal {K}} \pi _K=1$) on the collection of states belonging to $\mathcal {K}$.

Let $R \subseteq Q$ be the collection of all problems that received a correct response by a student, named the *response pattern*. A knowledge state $K \in \mathcal {K}$ is a latent construct underlying the response pattern *R* of a student; therefore, a perfect identity between *K* and *R* might not exist. Their relationship is established by an unrestricted latent class model, where the states $K \in \mathcal {K}$ are the latent classes.

In the BLIM, the probability *P*(*R*) of observing *R* in a randomly sampled student is defined as

1 $$\begin{aligned} P(R)=\sum _{K\in \mathcal {K}}{P(R|K)\pi _K}, \end{aligned}$$

where *P*(*R*|*K*) is the conditional probability of observing the response pattern *R* given that the knowledge state of the student is *K*.

The *response rule* assumption states that the conditional probability of obtaining a correct response to an item $q \in Q$, given a certain knowledge state $K \in \mathcal {K}$, depends on two parameters: $\beta _q \in [0,1)$, that is, the conditional probability of observing an incorrect answer to item *q* given that $q \in K$, and $\eta _q \in [0,1)$, that is, the conditional probability of observing a correct answer to item *q* given that $q \notin K$. The $\beta _q$ and $\eta _q$ parameters are called *careless error* and *lucky guess*, respectively.

Under the *response rule* assumption and the assumption of local independence of the item responses given the knowledge states, the conditional probability *P*(*R*|*K*) takes on the form

2 $$\begin{aligned} P(R|K)=\left[ \prod _{q \in K \setminus R} \beta _q\right] \left[ \prod _{q \in K \cap R} (1-\beta _q)\right] \left[ \prod _{q \in R \setminus K} \eta _q\right] \left[ \prod _{q \in Q \setminus (K \cup R)} (1-\eta _q)\right] . \end{aligned}$$

In this equation, each member of the product has the following meaning:

- $\prod _{q \in K \setminus R} \beta _q$ is the product of the probabilities of careless errors $\beta _q$ for the items belonging to the latent state *K*, but not to the observed response pattern *R*;
- $\prod _{q \in K \cap R} (1-\beta _q)$ is the product of the probabilities of not committing a careless error for each item belonging to both the latent state *K* and the observed pattern *R*;
- $\prod _{q \in R \setminus K} \eta _q$ is the product of the probabilities of lucky guesses $\eta _q$ for the items *q* contained in the response pattern *R*, but not in the latent state *K*;
- $\prod _{q \in Q \setminus (K \cup R)} (1-\eta _q)$ is the product of the probabilities of not committing lucky guesses for all items neither belonging to the pattern *R* nor to the state *K*.

Once again, an example could better clarify the crucial elements involved in the above definitions. Let now consider again the domain $Q_1=\{a,b,c,d,e\}$. Let, moreover, consider the case in which the response pattern $R=\{a,b,c\}$ is observed for a student whose knowledge state is $K=\{a,c,d\}$. In the case at hand, the student committed a careless error on item *d* which belongs to *K*, but not to *R*; she did not commit a careless error on items *a* and *c* which belong to both *K* and *R*; the student made a lucky guess on item *b*, which belongs to *R*, but not to *K*; finally, she made no lucky guess on item *e* which does belong to neither *R* nor *K*. Therefore, the conditional probability *P*(*R*|*K*) in the present example is:

$$\begin{aligned} P(\{a,b,c\}|\{a,c,d\})= \beta _d (1-\beta _a)(1-\beta _c)\eta _b(1-\eta _e). \end{aligned}$$

The $\beta _q$, $\eta _q$ and $\pi _K$ parameters of the BLIM can be estimated by maximum likelihood (ML) via the expectation–maximization (EM) algorithm (Stefanutti and Robusto 2009) or by minimum discrepancy (MD; Heller and Wickelmaier 2013). Moreover, methods for obtaining maximum likelihood estimates from data in which some responses are missing are available in the literature (Anselmi et al. 2016; de Chiusole et al. 2015), together with procedures for testing the invariance of the $\beta _q$ and $\eta _q$ parameters (de Chiusole et al. 2013). Some extensions of the model have been proposed for the assessment of learning processes, as the gain–loss model (GaLoM; Anselmi et al. 2012, 2017; de Chiusole et al. 2013; Robusto et al. 2010; Stefanutti et al. 2011), and a model for the treatment of skills dependence (de Chiusole and Stefanutti 2013).

Concerning the identifiability of the BLIM, it has been widely explored in recent years (e.g., Heller 2017; Spoto et al. 2012; 2013; Stefanutti et al. 2012, 2018; Stefanutti and Spoto 2020) providing a more in-depth understanding of the characteristics of the unidentifiable structures and providing useful tools for testing identifiability of the model. It has been shown that several important instances of knowledge structures happen to be forward or backward graded. Among them, there are the quasi-ordinal spaces and the linear orders. In these structures, the forward gradedness is established for all the items with no prerequisites, while backward gradedness is established for *non-background items* (i.e., all those items that are not included in the background knowledge of any other item). Any ordinal space contains a singleton for every item $q \in Q$ in which the structure is FG; respectively, any ordinal space contains a state of the form $Q \setminus \{q\}$ for every item $q \in Q$ in which the structure is BG. The same holds in any quasi-ordinal space in which the minimal and the maximal elements of the corresponding quasi-order are unique (Heller 2017). Any linear order contains one singleton for the minimum item in the order (which is the only item with no prerequisites) and a state $Q \setminus \{q\}$ for the maximum one (which is the only item not included in the background knowledge of any other item). As a consequence, any quasi-ordinal space corresponding to a linear order is FG in the minimum item and BG in the maximum one. In turn, this results in the unidentifiability of the $\eta$ parameter of the minimum item and of the $\beta$ parameter of the maximum one.

In the BLIM, the restriction $\beta _q + \eta _q < 1$, $q \in Q$, is usually a desirable property of the model’s parameters. This is a kind of “monotonicity” condition stating that the probability of failing an item *q* by a careless error should be strictly less than that of failing it because it is not mastered ($\beta _q < 1 - \eta _q$). Equivalently, it states that the probability of correctly solving the item because it is mastered must be strictly greater than that of guessing it ($\eta _q < 1 - \beta _q$).

For any item $q \in Q$, the error parameters $\beta _q$ and $\eta _q$ can be represented in matrix form:

$$\begin{aligned} E = \begin{pmatrix} 1-\eta _q &{} \eta _q \\ \beta _q &{} 1-\beta _q \\ \end{pmatrix} \end{aligned}$$

Indicating with $\pi _q$ the probability that *q* belongs to the state of a randomly sampled individual, and considering any probability vector $\pi = (1-\pi _q,\pi _q)^T$, the product $E^T\pi$ gives a column vector $(1-p_q,p_q)^T$, where

$$\begin{aligned} p_q&= \eta _q (1-\pi _q) + (1-\beta _q)\pi _q \\&= (1-\beta _q-\eta _q)\pi _q+\eta _q \end{aligned}$$

is the probability that *q* belongs to the individual’s response pattern. The term “monotonicity” refers to the fact that such probability is monotone increasing in $\pi _q$ if and only if $\beta _q < 1-\eta _q$. This type of monotonicity could be named *column monotonicity*, because the two terms of the inequality belong to the same column of the matrix *E*. Another, more restrictive type of monotonicity for the dichotomous BLIM is to require that $\beta _q < 1-\beta _q$ and $\eta _q<1-\eta _q$ for all $q \in Q$. Since both $\beta _q$ and $\eta _q$ are regarded as “error probabilities,” such inequalities follow the principle that an error should be less likely than a non-error. This last type of monotonicity will be named *row monotonicity*, because the two terms of the inequality belong to the same row of *E*. Then, it is easily verified that row monotonicity implies column monotonicity.

As far as the BLIM is concerned, all these considerations about monotonicity are rather straightforward. However, they are preparatory to a polytomous generalization of the BLIM, where the matrix *E* may have an arbitrary number of rows and columns. This is the subject matter of Sect. 3.

### Polytomous KST

The crucial assumption at the basis of the extension of KST to polytomous items is the possibility of scoring each item in the domain *Q* through *levels* in a set *L*. In this perspective, the classical dichotomous KST becomes the special case in which the cardinality of the set *L* is 2. This fundamental assumption implies the redefinition of a knowledge state as a function $K:Q \rightarrow L$ that assigns levels to items (Schrepp 1997; Stefanutti et al. 2020). Since the items of attitude and personality scales usually evaluate beliefs of individuals about themselves or the world, the states will be sometimes denoted as *belief states*, rather than knowledge states.

A *polytomous structure* is any nonempty subset $\mathcal {K}\subseteq L^Q$, where $L^Q$ is the collection of all the mappings $K: Q \rightarrow L$. The mappings in $L^Q$ are partially ordered by the *pointwise* order $\sqsubseteq$ such that, given any two mappings $K_1,K_2 \in L^Q$,

$$\begin{aligned} K_1 \sqsubseteq K_2 \text { iff } K_1(q) \leqslant K_2(q) \text { for all } q \in Q. \end{aligned}$$

In a first generalization of KST, Schrepp (1997) proposed a reformulation of the main deterministic elements of the theory in order to have available, for each item $q \in Q$, more than two answer alternatives taken from a linearly ordered set $(L,\leqslant )$. Each of these alternatives indicates, in an ordinal way, the level of solution of a specific item *q*. In this perspective, as mentioned above, a knowledge state becomes a mapping from *Q* to *L*, that is, it is redefined as a way to assign levels of *L* to items in *Q*. In the approach proposed by Schrepp, this fundamental assumption allows for the generalization of the concepts of knowledge structure, knowledge space, and quasi-ordinal knowledge space. Furthermore, the closure properties to be satisfied in order to have a one-to-one correspondence between structures and surmise functions and relations in the polytomous case are stronger than in the dichotomous one.

Schrepp’s approach is not the only attempt to generalize KST to the polytomous case. In fact, from a quite different perspective, Bartl and Belohlavek (2011) proposed an extension of KST which assumes that a knowledge state is a fuzzy (graded) set, with degrees representing levels to which an individual has mastered the items. These two approaches make different assumptions on the set *L* of levels. In fact, in the Schrepp’s proposal, *L* is any linearly ordered set. In the Bartl and Belohlavek’s approach, *L* is a *complete residuated lattice*. Although this last assumption allows Bartl and Belohlavek’s approach to be suitable for the case in which *L* is infinite, it implies the existence of a kind of “concatenation” operator $\otimes$ among levels in *L* such that, given any two levels $a,b \in L$, $a \otimes b$ is also in *L*. This last assumption may not correctly characterize the ordinal nature of the elements in *L*, which are typical in polytomous KST applications (mostly social and behavioral sciences).

In the generalization proposed by Stefanutti et al. (2020), the set *L* of levels is assumed to be any linearly ordered complete lattice. This assumption makes the approach by Stefanutti and colleagues more restrictive than that by Schrepp, but still less restrictive than that by Bartl and Belohlavek. The approach to the polytomous extension of KST we refer to in this article is the one proposed by Stefanutti et al. (2020) for *L* finite.

## The Polytomous Local Independence Model

The BLIM, with all of its assumptions, can be generalized to polytomous structures and response patterns with no further assumptions other than those admitting more than two response alternatives. Let *Q* and *L* be finite sets, and let $R \in L^{Q}$ be the pattern of the observed responses of an individual to the set *Q* of items. If the state of this individual is $K \in \mathcal {K}$, then we allow that *R* and *K* may differ to some extent, thus dissociating an *observed response* *R*(*q*) from a *latent response* *K*(*q*) to an item $q \in Q$. Any difference between the two may be imputable to random error.

For $q \in Q$, let $\mathbf {R}_q$ and $\mathbf {K}_q$ be random variables having realizations in *L* and representing the observed and latent responses to item *q*, respectively. Furthermore, let $\mathbf {R}= \{\mathbf {R}_q\}_{q \in Q}$ be a random vector with realizations in $L^{Q}$ and $\mathbf {K}= \{\mathbf {K}_q\}_{q \in Q}$ be a random vector with realizations in $\mathcal {K}$. The random vectors $\mathbf {R}$ and $\mathbf {K}$ represent, respectively, the observed response pattern and the latent state of a randomly sampled individual.

The *response rule* of the BLIM is generalized as follows. Let $q \in Q$ be any item, $i,j \in L$ be any two levels in *L*, and $K \in \mathcal {K}$ be any state, then

$$\begin{aligned} K(q) = i \implies P(\mathbf {R}_q=j|K)=\epsilon _{q}(i,j), \end{aligned}$$

with $\epsilon _{q}: L^2 \rightarrow (0,1)$. This assumption states that the conditional probability of observing response *j* to item *q* depends on the response itself, the level *K*(*q*) assigned by the latent state *K* to *q* and nothing else.

The BLIM’s local independence assumption is generalized as follows. For $R \in L^Q$ and $K \in \mathcal {K}$, the responses to the items are locally independent given state *K*:

$$\begin{aligned} P(\mathbf {R}= R|\mathbf {K}= K) = \prod _{q \in Q} P(\mathbf {R}_q = R(q)|\mathbf {K}=K). \end{aligned}$$

By combining these two assumptions, we obtain the first equation of the PoLIM:

$$\begin{aligned} P(\mathbf {R}=R|K) = \prod _{q \in Q} \epsilon _q(K(q),R(q)). \end{aligned}$$

The marginal probability of a response pattern is obtained, as in the standard BLIM, by

$$\begin{aligned} P(R) = \sum _{K \in \mathcal {K}}P(R|K)\pi _K \end{aligned}$$

where $\pi _K \in (0,1)$ is the probability of state $K \in \mathcal {K}$. This gives the equation

$$\begin{aligned} P(R) = \sum _{K \in \mathcal {K}}\prod _{q \in Q} \epsilon _q(K(q),R(q))\pi _K. \end{aligned}$$

Given the two constraints (which hold true even in the dichotomous BLIM)

3 $$\begin{aligned} \sum _{j \in L} \epsilon _q(i,j)=1, q \in Q, i \in L \end{aligned}$$

and

$$\begin{aligned} \sum _{K \in \mathcal {K}} \pi _K = 1, \end{aligned}$$

the model has $|Q||L|(|L|-1)+|\mathcal {K}|-1$ free parameters.

The dichotomous BLIM is the special case of the PoLIM in which $L=\{0,1\}$. In fact, given the constraint in (3), it suffices to set $\epsilon _q(1,0)=\beta _q$ and $\epsilon _q(0,1)=\eta _q$ for each item $q \in Q$.

In the general case $L = \{l_0,l_1,\ldots ,l_n\}, n \ge 1$, the $\epsilon _q$ functions are represented by squared matrices of the form

$$\begin{aligned} E_q = \begin{pmatrix} \epsilon _q(l_0,l_0) &{} \epsilon _q(l_0,l_1) &{} \ldots &{} \epsilon _q(l_0,l_n) \\ \epsilon _q(l_1,l_0) &{} \epsilon _q(l_1,l_1) &{} \ldots &{} \epsilon _q(l_1,l_n) \\ \vdots &{} \vdots &{} &{} \vdots \\ \epsilon _q(l_n,l_0) &{} \epsilon _q(l_n,l_1) &{} \ldots &{} \epsilon _q(l_n,l_n) \end{pmatrix}. \end{aligned}$$

The off-diagonal elements of $E_q$ are error probabilities. If the levels in *L* are totally ordered, with $l_0 \preceq l_1 \preceq \cdots \preceq l_n$, then the probabilities belonging to the lower triangular matrix of $E_q$ can be regarded as “underrates” of the true level, whereas the probabilities belonging to the upper triangular matrix can be regarded as “overrates” of the true level.

### Monotonicity and Other Restrictions

The model described in the previous section does not impose any restriction on the $\epsilon _q(i,j)$ parameters, other than they must sum up to 1 across the levels $j \in L$ for any $q \in Q$ and any $i \in L$. It says nothing about the interplay between these probability values and the levels in *L*.

As observed in Sect. 2.2, two distinct forms of monotonicity are possible for the parameters of the dichotomous BLIM: row and column monotonicity. At least the less restrictive of the two forms (i.e., column monotonicity) is required for having consistent assessment. We explore in this section how such notions of monotonicity can be transferred from the dichotomous to the polytomous case. When moving to a polytomous framework, there is no unique way of generalizing these two types of monotonicity. For instance, there are at least three different ways of generalizing row monotonicity: (1) If the state *K* assigns level $i \in L$ (call it the “true level”) to an item $q \in Q$, then it is highly reasonable that, in an assessment, this value will be the most likely to be observed; (2) The restriction could even be stronger, by requiring that the overall probability of observing *any* “false level” is less than that of observing the “true level”; (3) Moreover, it makes sense that, as the “distance” of the observed level from the true one increases, the probability of the observed level decreases. This type of assumption, for instance, is at the core of the statistical theory of error in the classical (true score) test theory. In applications with continuous (latent) variables, it is often assumed that error is distributed normally around the true score. A consequence of this assumption is that the probability density of an error decreases as the distance from the true score increases. A rather general definition of this type of monotonicity, which applies to either finite or infinite countable sets, is as follows. We recall that a metric on a set *X* is any function $\delta :X^2 \rightarrow \mathbb {R}$ satisfying the following properties for all $x,y,z \in X$:

(i) identity of indiscernibles: $\delta (x,y) = 0 \iff x=y$;
(ii) symmetry: $$\delta (x,y) = \delta (y,x)$$;
(iii) triangle inequality: $\delta (x,z) \le \delta (x,y)+\delta (y,z)$.

Furthermore, it is a consequence of (i), (ii), and (iii) that $d(x,y) \ge 0$ for all $x,y \in X$.

Let $\delta : L^2 \rightarrow \mathbb {R}$ be a metric on the set *L* of levels. A function $f: L^2 \rightarrow \mathbb {R}$ is said to be $\delta$-*monotone* if the double implication

4 $$\begin{aligned} \delta (i,j) < \delta (i,k) \iff f(i,j) > f(i,k) \end{aligned}$$

holds true for all $i,j,k \in L$. Furthermore, the function *f* is named $\delta$*-half-monotone* if Condition (4) holds when confined to triples $i,j,k \in L$ such that either $i \preceq \min \{j,k\}$ or $\max \{j,k\} \preceq i$ holds (i.e., *j* and *k* are both predecessors and successors of *i*). It is an immediate consequence that $\delta$-monotonicity implies $\delta$-half-monotonicity, whereas the opposite implication need not be true.

To give an example, let $L=\{a,b,c,d\}$ be a set of four levels linearly ordered by $\preceq$, with $a \preceq b \preceq c \preceq d$. Let, moreover, $\delta _1:L^2 \rightarrow \mathbb {R}$ be a distance function defined by the following $|L| \times |L|$ symmetric matrix:

$$\begin{aligned} \delta _1 = \begin{pmatrix} 0&{} 1&{} 3&{} 5\\ 1&{} 0&{} 2&{} 3\\ 3&{} 2&{} 0&{} 1\\ 5&{} 3&{} 1&{} 0\\ \end{pmatrix}, \end{aligned}$$

whose each entry represents a single pair (*i*, *j*) of levels in *L*. Thus, for instance, rows 1, 2, 3 and 4 represent levels *a*, *b*, *c* and *d*, respectively, so that $\delta _1(a,b)=1$, $\delta _1(a,c)=3$, $\delta _1(a,d)=5$ and so on. Define the function $f_1:L^2 \rightarrow \mathbb {R}$ by the following square matrix:

$$\begin{aligned} f_1 = \begin{pmatrix} 20&{} 18&{} 10&{} 5\\ 10&{} 40&{} 5 &{} 8\\ 7 &{} 8 &{} 15&{} 4\\ 1 &{} 2 &{} 3 &{} 4\\ \end{pmatrix}, \end{aligned}$$

so that, for instance, $f_1(a,a)=20$, $f_1(a,b)=18$, $f_1(a,c)=10$, and so on. Then, $f_1$ respects $\delta _1$-half-monotonicity, but not $\delta _1$-monotonicity. For testing $\delta _1$-monotonicity of function $f_1$, condition (4) has to be tested for each of the $4^3 = 64$ triples (*i*, *j*, *k*) of levels in *L*. In particular, if the condition is tested for the triple (*c*, *d*, *a*), one obtains $\delta _1(c,d)=1<\delta _1(c,a)=3$, but $f_1(c,d)=4<f_1(c,a)=7$. This falsifies $\delta _1$-monotonicity of $f_1$. On the other side, if only triples satisfying $i \preceq \min \{j,k\}$ or $\max \{j,k\} \preceq i$ are considered (there are 44 in the whole), then condition (4) is always satisfied, confirming $\delta _1$-half-monotonicity of $f_1$.

#### Monotonicity in Unordered Sets of Levels

The specific form taken by the $\delta$-monotonicity condition much depends on the chosen metric $\delta$ which, in turn, depends on the properties of the set *L* of levels. When *L* is a finite and unordered set (e.g., in multiple choice items), the only meaningful metric is the Hamming distance which, for $i,j \in L$, is defined as

5 $$\begin{aligned} \delta _H(i,j) = {\left\{ \begin{array}{ll} 0 &{} \text {if } i = j, \\ 1 &{} \text {if } i \ne j. \end{array}\right. } \end{aligned}$$

The form that $\delta$-monotonicity takes when the metric is $\delta _H$ is named *modality*. Thus, for $q \in Q$, the function $\epsilon _q$ respects modality if and only if it is $\delta _H$-monotone. The term “modality” stems from the observation that $\delta _H$-monotonicity only requires that the inequality $\epsilon _q(i,i) > \epsilon _q(i,j)$ stays true for all $i,j \in L$, meaning that the true level *i* is modal. We say that the PoLIM repects modality if $\epsilon _q$ respects it for all $q \in Q$. We observe that modality is preserved after permutations that leave unchanged the probability of the true value.

#### Monotonicity in Ordered Sets of Levels

If *L* is totally ordered by $\preceq$, then both the Hamming and a discrete version of the Manhattan distance can be determined between any two levels $i,j \in L$. The discrete Manhattan distance between any two levels $i,j \in L$ is defined as

6 $$\begin{aligned} \delta _M(i,j)&= |(i^\downarrow \setminus j ^\downarrow )\cup (j^\downarrow \setminus i^\downarrow )|\nonumber \\&= abs(|i^\downarrow | - |j^\downarrow |), \end{aligned}$$

where, for any $l \in L$, $l^\downarrow = \{j \in L: j \preceq l\}$ is the down set of *l* in the partially ordered set $(L,\preceq )$. If $i \preceq j$, then the discrete Manhattan distance counts the number of levels greater than *i* that are less or equal to *j* in the totally ordered set *L*. For instance, if $L=\{a,b,c,d\}$, with $a \preceq b \preceq c \preceq d$, then $\delta _M(a,b)=1, \delta _M(a,c)=2, \delta _M(a,d)=3,$ and so on.

The form that $\delta$-monotonicity (respectively, $\delta$-half-monotonicity) takes when $\delta$ is the Manhattan distance is simply named *monotonicity* (respectively, *half-monotonicity*). Thus, for $q \in Q$, the function $\epsilon _q$ respects monotonicity if and only if it is $\delta _M$-monotone. We further say that the PoLIM respects monotonicity if $\epsilon _q$ respects it for all $q \in Q$. Monotonicity is preserved after strictly increasing transformations.

#### Order Respecting Metrics and $\delta$-Monotonicity

A metric that respects the order on the levels is named an “order respecting metric.” Given the totally ordered set $(L,\preceq )$ of levels, call $\delta :L^2 \rightarrow [0,\infty )$ an *order respecting metric* if it is a metric that satisfies the condition

7 $$\begin{aligned} i \prec j \prec k \implies \delta (i,k) > \max \{\delta (i,j),\delta (j,k)\}. \end{aligned}$$

for all $$i,j,k \in L$$.

The form of $\delta$-monotonicity may differ from one order respecting metric to another, in the sense that, given two order respecting metrics $\delta _1: L^2 \rightarrow [0,\infty )$ and $\delta _2: L^2 \rightarrow [0,\infty )$, a function $f: L^2 \rightarrow (0,1)$ may be $\delta _1$-monotone while being not $\delta _2$-monotone. That is, *f* is not necessarily $\delta$-monotone under any arbitrary order respecting metric $\delta$. To give an example, let $L = \{a,b,c\}$ be a set of three levels, with $a \preceq b \preceq c$, and consider the two metrics $\delta _1: L^2 \rightarrow [0,\infty )$ and $\delta _2:L^2 \rightarrow [0,\infty )$ defined as follows:

$$\begin{aligned}&\delta _1(a,b) = 1,\quad \delta _1(a,c) = 3, \quad \delta _1(b,c) = 2,\\&\delta _2(a,b) = 3,\quad \delta _2(a,c) = 4, \quad \delta _2(b,c) = 2. \end{aligned}$$

It is easily verified that both metrics $\delta _1$ and $\delta _2$ respect the order $\preceq$ on the set of levels. Then, the function $f_2:L^2 \rightarrow (0,1)$ such that

$$\begin{aligned} f_2(b,a) = .2, \quad f_2(b,c) = .1, \end{aligned}$$

is $\delta _1$-monotone, but it is not $\delta _2$-monotone.

Concerning $\delta$-half-monotonicity, instead, the following theoretical result holds.

##### Proposition 1

A function $f: L^2 \rightarrow \mathbb {R}$ is $\delta$-half-monotone for every order respecting metric $\delta$ if and only if it is $\delta ^*$-monotone for some order respecting metric $\delta ^*$.

##### Proof

Let $\delta$ and $\delta ^*$ be any two order respecting metrics for $(L,\preceq )$, and let $f:L^2 \rightarrow \mathbb {R}$ be a $\delta ^*$-monotone function. Suppose then that *f* is not $\delta$-half-monotone. In that case, there must exist a triple $(i,j,k) \in L^3$ for which all the following three conditions hold true: (1) $\delta (i,j) < \delta (i,k)$; (2) $f(i,j) < f(i,k)$; (3) $i \preceq \min \{j,k\}$ or $\max \{j,k\} \preceq i$. Since *f* is $\delta ^*$-monotone, from $f(i,j) < f(i,k)$, we obtain $\delta ^*(i,j) > \delta ^*(i,k)$. Suppose $i \preceq \min \{j,k\}$ holds true. Then, it follows from $\delta ^*(i,j) > \delta ^*(i,k)$ that $i \preceq k \prec j$. But then, $\delta (i,j) < \delta (i,k)$ contradicts the order preserving property of $\delta$. Suppose now $\max \{j,k\} \preceq i$ holds. Then, it follows from $\delta ^*(i,j) > \delta ^*(i,k)$ that $j \prec k \preceq i$. But then, again, $\delta (i,j) < \delta (i,k)$ contradicts the order preserving condition. We thus conclude that *f* must be $\delta$-half-monotone. $\square$

Thus, $\delta$-half-monotonicity depends on the order on the levels in *L*, but not on the order of the distances among the levels. In practice, this means, for instance, that if one chooses as a metric the Manhattan distance $\delta _M$, then any $\delta _M$-monotone function is a $\delta$-half-monotone function for every order respecting metric $\delta$. Thus, it is not necessary to know the true underlying metric $\delta$ to conclude that a given conditional probability function $\epsilon _q$ is $\delta$-half-monotone and thus respects the order on the levels.

An additional condition that may turn out to be interesting is named *overall error*, and it states that

8 $$\begin{aligned} \epsilon _q(i,i) > \sum _{j \in L \setminus \{i\}} \epsilon _q(i,j) \end{aligned}$$

for all $q \in Q$ and all $i \in L$. According to this condition, not only the true level is the more probable one (modality), but its probability is larger than the sum of the probabilities of all the other levels. In fact, it follows from this condition that $\epsilon _q(i,i)>1/2$.

So far, only row monotonicity conditions were examined. The $\delta$-monotonicity condition is easily adapted to column monotonicity: Just set $f(i,j)=\epsilon _q(j,i)$ in Eq. (4). With this substitution, each of the row monotonicity conditions considered above can be easily translated into a column monotonicity condition.

It is always possible to make posterior tests of such conditions on the parameter estimates of the unrestricted PoLIM. Otherwise, the monotonicity constraints can be directly incorporated into the model.

### A PoLIM with Monotonicity Constraints

A version of the PoLIM that incorporates monotonicity conditions is described in this section. More precisely, let $\delta$ be any order respecting metric for $(L,\preceq )$. A version of the PoLIM that respects the assumption under which, for every item $q \in Q$, the function $\epsilon _q$ is $\delta$-half-monotonic, is developed. The $\delta$-half-monotonicity condition requires that

$$\begin{aligned} \delta (i,j)<\delta (i,k) \iff \epsilon _q(i,j)>\epsilon _q(i,k) \end{aligned}$$

for all $i,j,k \in L$ such that $i \preceq \min \{j,k\}$ or $\max \{j,k\} \preceq i$, which entails a system of linear inequalities involving the model’s $\epsilon _q(i,j)$ parameters. If the aim is estimating such parameters, then there is no trivial variant of the expectation–maximization algorithm that incorporates linear inequalities among the parameters of a model. However, one can see if it is possible to resort to some suitable reparameterization of the constrained model to an equivalent one where there are no inequality constraints among the parameters. This is the route followed here. In particular, an equivalent model is obtained, where inequalities of the form $x < y$ are replaced by inequalities of the form $0< x < 1$ and $0< y < 1$. For lightening notation, lowercase letters like *i*, *j*, *k*, *l* denote integer subscripts, instead of levels in *L*.

Let $L = \{\ell _0,\ell _1,\ldots ,\ell _n\}$ (so that the number of levels is $n+1$) and, for $q \in Q$, let $\mathbf {R}_q$ and $\mathbf {K}_q$ be random variables, whose realizations are the levels in *L*. The random variable $\mathbf {R}_q$ represents the observed response to an item *q*, whereas $\mathbf {K}_q$ represents the “latent response” to *q*. Given any two indexes $i,j \in \{0,1,\ldots ,n\}$ such that $i \le j < n$, let $\omega _{qij}$ denote the ratio between the two conditional probabilities $P(\mathbf {R}_q=\ell _j|\mathbf {K}_q=\ell _i)$ and $P(\mathbf {R}_q=\ell _{j+1}|\mathbf {K}_q=\ell _i)$, that is:

9 $$\begin{aligned} \omega _{qij}=\frac{\epsilon _q(i,j+1)}{\epsilon _q(i,j)}= \frac{P(\mathbf {R}_q=\ell _{j+1}|\mathbf {K}_q=\ell _i)}{P(\mathbf {R}_q=\ell _j|\mathbf {K}_q=\ell _i)}, \end{aligned}$$

whereas, for $0 < j \le i$, let $\upsilon _{qij}$ denote the ratio:

10 $$\begin{aligned} \upsilon _{qij}= \frac{\epsilon _q(i,j-1)}{\epsilon _q(i,j)}= \frac{P(\mathbf {R}_q=\ell _{j-1}|\mathbf {K}_q=\ell _i)}{P(\mathbf {R}_q=\ell _j|\mathbf {K}=\ell _i)}. \end{aligned}$$

The $\delta$-half-monotonicity assumption is satisfied if and only if the rates $\omega _{qij}$ and $\upsilon _{qij}$ are in the open interval (0, 1). Confining the values of the two parameter types to this interval, the parameter $\omega _{qij}$ can be regarded as a rate of decay of the probability of “overrating” item *q* as the overrate increases, whereas the parameter $\upsilon _{qij}$ is regarded as a rate of decay of the probability of “underrating” item *q* as the underrate decreases. It follows from Eqs. (9) and (10) that, for any two indexes $i \le j < n$,

$$\begin{aligned} \epsilon _q(i,j+1) = \omega _{qij} \epsilon _q(i,j) \end{aligned}$$

and for any two indexes $0 < j \le i$

$$\begin{aligned} \epsilon _q(i,j-1) = \upsilon _{qij} \epsilon _q(i,j). \end{aligned}$$

Therefore, all probabilities $\epsilon _q(i,j)$ can be expressed as functions of the diagonal probabilities $\epsilon _{qi} := \epsilon _q(i,i)$ and the two rates $\omega _{qij}$ and $\upsilon _{qij}$:

11 $$\begin{aligned} \epsilon _q(i,j) = {\left\{ \begin{array}{ll} \epsilon _{qi}\prod _{k=j}^{i-1}\upsilon _{qik} &{} \text {if } i > j, \\ \epsilon _{qi} &{} \text {if } i=j, \\ \epsilon _{qi}\prod _{k=i+1}^j\omega _{qik} &{} \text {if } i < j. \end{array}\right. } \end{aligned}$$

Plugging this last equation into the equality constraints

$$\begin{aligned} \sum _{l=0}^{n} \epsilon _q(i,l) = 1, \quad \forall i \in \{0,1,\ldots ,n\} \end{aligned}$$

we obtain that, for all $i=0,1,\ldots n$,

$$\begin{aligned} \epsilon _{qi} + \sum _{l=0}^{i-1} \epsilon _{qi}\prod _{k=l}^{i-1}\upsilon _{qik} + \sum _{l=i+1}^{n}\epsilon _{qi}\prod _{k=i+1}^l\omega _{qik} = 1, \end{aligned}$$

and, solving for the diagonal probability $\epsilon _{qi}$,

$$\begin{aligned} \epsilon _{qi} = \left( 1+\sum _{l=0}^{i-1} \prod _{k=l}^{i-1}\upsilon _{qik} + \sum _{l=i+1}^{n}\prod _{k=i+1}^l\omega _{qik}\right) ^{-1}. \end{aligned}$$

Finally, by substituting $\epsilon _{qi}$ with the right-hand side of this last equation into (11), one obtains the following equation, where $\epsilon _q(i,j)$ is a function of the two parameter types $\omega$ and $\upsilon$:

12 $$\begin{aligned} \epsilon _q(i,j) = {\left\{ \begin{array}{ll} \displaystyle {\prod _{k=j}^{i-1}\upsilon _{qik}\left( 1+\sum _{l=0}^{i-1} \prod _{k=l}^{i-1}\upsilon _{qik} + \sum _{l=i+1}^{n}\prod _{k=i+1}^l\omega _{qik}\right) ^{-1}} &{} \text {if } i > j, \\ \displaystyle {\left( 1+\sum _{l=0}^{i-1} \prod _{k=l}^{i-1}\upsilon _{qik} + \sum _{l=i+1}^{n}\prod _{k=i+1}^l\omega _{qik}\right) ^{-1}} &{} \text {if } i=j, \\ \displaystyle {\prod _{k=i+1}^j\omega _{qik}\left( 1+\sum _{l=0}^{i-1} \prod _{k=l}^{i-1}\upsilon _{qik} + \sum _{l=i+1}^{n}\prod _{k=i+1}^l\omega _{qik}\right) ^{-1}} &{} \text {if } i < j. \end{array}\right. } \end{aligned}$$

Therefore, a PoLIM with monotonicity constraints is obtained via a reparameterization into a model with parameters $\omega _{qij},\upsilon _{qij} \in (0,1)$ for each item $q \in Q$, and pairs of levels $i,j \in L$.

To exemplify, let $L=\{\ell _0,\ell _1,\ell _2,\ell _3,\ell _4\}$ be the set of levels, with $\ell _i \prec \ell _{i+1}, i \in \{0,1,2,3\}$. For some $q \in Q$, suppose that

$$\begin{aligned} \upsilon _{q20}=\frac{2}{10}, \quad \upsilon _{q21}=\frac{1}{10}, \quad \omega _{q23}=\frac{3}{10}, \quad \omega _{q24}=\frac{5}{10}. \end{aligned}$$

For the case $i=2$, the probabilities $\epsilon _q(i,j), j \in \{0,1,\ldots ,4\}$ are obtained from these four rates, by an application of the formulas in Eq. (12). Starting with the diagonal element $\epsilon _q(2,2)$, one has:

$$\begin{aligned} \epsilon _q(2,2)&= \left( \upsilon _{q20}\upsilon _{q21}+\upsilon _{q21}+1+\omega _{q23}+\omega _{q23}\omega _{q24}\right) ^{-1} \\&= \left( \frac{2}{10}\cdot \frac{1}{10}+\frac{1}{10}+1+\frac{3}{10}+\frac{3}{10}\cdot \frac{5}{10}\right) ^{-1} = \frac{100}{157}. \end{aligned}$$

Then, one has

$$\begin{aligned} \epsilon _q(2,0)&= \epsilon _q(2,2)\upsilon _{q20}\upsilon _{q21} = \frac{2}{157},\\ \epsilon _q(2,1)&= \epsilon _q(2,2)\upsilon _{q21} = \frac{10}{157},\\ \epsilon _q(2,3)&= \epsilon _q(2,2)\omega _{q23} = \frac{30}{157},\\ \epsilon _q(2,4)&= \epsilon _q(2,2)\omega _{q23}\omega _{q24} = \frac{15}{157}. \end{aligned}$$

Thus, we have:

$$\begin{aligned} \epsilon _q(2,0)+\epsilon _q(2,1)+\epsilon _q(2,2)+\epsilon _q(2,3)+\epsilon _q(2,4)=1 \end{aligned}$$

and

$$\begin{aligned} \epsilon _q(2,0)<\epsilon _q(2,1)<\epsilon _q(2,2)>\epsilon _q(2,3)>\epsilon _q(2,4), \end{aligned}$$

that is, the set $\{\epsilon _q(2,j)\}_{j=0}^4$ is a probability distribution that respects $\delta$-half-monotonicity. It is also noticeable that $\delta$-monotonicity is not respected in this case. In fact, for instance, $\epsilon _q(2,4) > \epsilon _q(2,1)$.

### Parameter Estimation

The most frequently used procedures for estimating the parameters of the BLIM are by maximum likelihood (ML) and by minimum discrepancy (MD). Both of them have been extended for estimating the parameters of the PoLIM. Only ML has been extended for estimating the parameters of the PoLIM with monotonicity constraints.

ML estimation is accomplished via an adaptation of the EM algorithm developed by Stefanutti and Robusto (2009), whereas MD estimation is obtained as a generalization of the minimum discrepancy method developed by Heller and Wickelmaier (2013). The main differences between the two procedures are:

(i) EM maximizes the likelihood of the data, given the model parameters, whereas MD minimizes a measure of discrepancy between the data and the states in the structure;
(ii) MD is based on the (rather strong) assumption that a response pattern has a positive probability of being “generated” by a certain state only if this last is at minimum distance from it. This assumption does not apply in the EM, where any response pattern can be generated by any state with positive probability;
(iii) the MD procedure requires to assume the form of the metric that has to be minimized (e.g., Hamming, Manhattan, Euclidean distance, etc.). Such an assumption is not needed in the EM algorithm;
(iv) the EM is an iterative algorithm, whereas the MD provides analytic formulas for computing the parameter estimates. As such, it requires no iterations;
(v) the EM algorithm can be adapted for estimating the PoLIM with monotonicity (inequality) constraints; it is not obvious how this can be done with the MD method.

A detailed description of both the EM and the MD is found in Appendices A.1, A.2 and A.3. They have been implemented in MATLAB and are available upon request to the authors.

## Simulation Study

The aim of the study was to check the parameter recovery capability of the EM and the MD algorithms with respect to the PoLIM, when the (row) half-monotonicity assumption is respected or not in the data. For the EM, both the constrained and unconstrained estimation procedures were considered. For the MD, both the Hamming and the Manhattan distances were used for computing the distances between the response patterns and the states.

### Simulation Design, Data Set Generation and Methods

Let *Q* be a fixed set of 10 items and $L=\{0,1,2,3\}$ be a linearly ordered set of response categories of the items $q \in Q$. Let $\bot _{|Q|}$ be the |*Q*|-tuple of all zeros, and $\top _{|Q|}=(3,3,\cdots ,3)$ be the maximum |*Q*|-tuple. A structure $\mathcal {K}$ of 1,000 states was obtained by computing $\{\bot _{|Q|},\top _{|Q|}\} \cup \mathcal {P}$, where $\mathcal {P}$ was generated at random, using a sampling without replacement on the collection $L^{|Q|} \setminus \{\bot _{|Q|},\top _{|Q|}\}$. In this random structure, the proportion of states assigning to an item $q \in Q$ a given level $l \in L$ is about $1/|L| = .25$. The uniform probability distribution was assumed on $\mathcal {K}$.

Two different scenarios have been considered. In both of them, the model that generated the data was the PoLIM. What varied between the two was the particular restriction used for generating the PoLIM’s parameters $\epsilon _{q}(i,j)$. In the former scenario, only the overall error condition (Eq. (8)) held. In the latter scenario, both the overall error and the monotonicity (Eq. (12)) conditions held.

The procedure used for generating the $\epsilon _{q}(i,j)$ parameters consisted in two steps. The first step was the generation of the “true positive” probability $\epsilon _{q}(i,i)$ (i.e., the probability that the level observed for *q* in the response pattern is equal to the level of *q* in the state). For each item $q \in Q$ and each level $i \in L$, $\epsilon _{q}(i,i)$ was randomly extracted from the uniform distribution in the interval [*m*, 1), with $m >1/2$. For all $q \in Q$, the same values of $\epsilon _{q}(i,i)$ were used in the two scenarios. In the second step, the $\epsilon _q(i,j)$, with $i \ne j$, were generated differently in the two scenarios. In the former scenario, they were extracted at random from the uniform distribution in the interval $(0,1-\epsilon _q(i,i)]$, and then, they were normalized to sum up to $1-\epsilon _q(i,i)$. In the latter scenario, in which both the overall error and the monotonicity (Eq. (12)) conditions held, the same values of the $\epsilon _q(i,j)$ generated in the former scenario were used, with the difference that they were ordered in size for respecting the monotonicity. For example, if the item *q* takes on level 0 in the state, then $\epsilon _q(i,1) \ge \epsilon _q(i,2) \ge \epsilon _q(i,3)$.

In each scenario, 100 different samples of size 2,000 and 5,000 were generated under three different conditions. What varied across conditions was the value of *m* used for generating the $\epsilon _q(i,i)$ parameters that was $m \in \{.75,.85,.95\}$. These values have been chosen with the rationale of simulating data by using not too high error rates. Referring to previous studies in which other models (e.g., the BLIM, the DINA, or other KST and CDM models) were applied, values for item error probabilities between .05 and .25 are rather typical.

The structure $\mathcal {K}$ and the probability distribution on the states were held fixed across all conditions of both scenarios.

A total number of $2 \times 3 \times 100 =600$ random data sets were generated. In each condition of each scenario, the PoLIM was applied to the simulated data by using four different procedures for the parameter estimation, that is,

(i) maximum likelihood via the EM algorithm imposing no constraint;
(ii) maximum likelihood via the EM algorithm imposing the monotonicity constraint on the error parameters;
(iii) minimum discrepancy based on the Hamming distance (HD-MD);
(iv) minimum discrepancy based on the Manhattan distance (MD-MD).

In particular, in both the EM algorithms, the initial guesses of the model parameters were 1/|*L*| for the $\epsilon _q(i,j)$ parameters and $1/|\mathcal {K}|$ for the $\pi _K$ probabilities of the states.

The parameter recovery of the PoLIM estimated with the four different estimation methods was analyzed. It is important to underline that the parameter recovery of a probabilistic model can be analyzed correctly only if all of its parameters are identifiable. An empirical way for testing the identifiability of the parameters of a probabilistic model consists in: (i) estimating the parameters a large number of times on the same data, using different starting points, and (ii) checking the variability of the estimates that yield the largest and identical likelihoods. If the variability of the estimates of all parameters is small (e.g., in case of a probability, if the standard deviation is less than $10^{-3}$), then it is plausible that the parameters of the model are identifiable.

This empirical test was performed for testing the identifiability of the unconstrained and the constrained PoLIM parameters. Both versions of the model were estimated 50 times via the corresponding EM algorithm, by using one of the simulated samples. The log-likelihoods of the 50 fitted models were very close to each other for both models (i.e., their values had a range of $1.93 \times 10^{-4}$ for that unconstrained and of $5.59 \times 10^{-4}$ for that constrained). Moreover, the maximum standard error of the estimates was $4.43 \times 10^{-7}$ and $2.47 \times 10^{-7}$, respectively, for the unconstrained and the constrained model. All of this may suggest that the parameters are identifiable in both cases. Of course, this result should be taken as provisional, until some formal test of (local) identifiability of the PoLIM becomes available.

### Results and Discussion

**Fig. 1:**
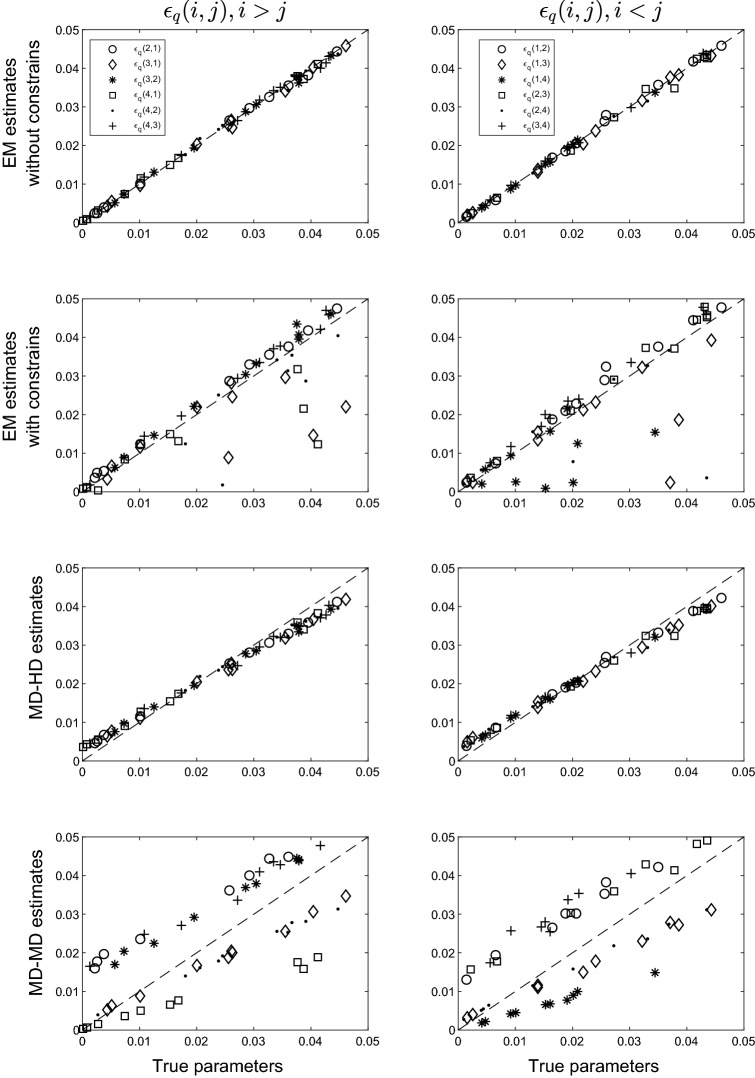
Parameter recovery of the PoLIM obtained by using the EM algorithm without constraints and with constraints, and by using the MD-HD and the MD-MD, when only the overall error condition holds in the data

Results for the $N=$ 2,000 case are presented in this section. Results for $N=$ 5,000 do not substantially differ from those for $N=$ 2,000; thus, they are provided in the form of supplementary material. Figure 1 shows the parameter recovery of the $\epsilon _q(i,j)$ PoLIM parameters in the first condition (i.e., $\epsilon _{q}(i,i) = .95$) of the first scenario, in which only the overall error condition holds in the simulated data.

In each panel of the figure, true parameter values are on the *x*-axis, estimated parameter values are on the *y*-axis, and the dotted line indicates that $x=y$. The *y*-axis labels indicate the parameter estimation method among the unconstrained and constrained EM, HD-MD and MD-MD. Panels to the left of the figure display the results of the $\epsilon _{q}(i,j)$ parameters when $i > j$ (i.e., $K(q)>R(q)$) and panels on the right that of the $\epsilon _{q}(i,j)$ parameters when $i<j$ (i.e., $K(q)<R(q)$). The standard errors of the estimates are reported in Table 1, in which their average and maximum values are given for each of the four estimation methods.

**Table 1:** Average (columns 2, 4 and 6) and maximum (columns 3, 5 and 7) standard errors of the PoLIM’s parameter estimates obtained for the four estimation methods unconstrained EM, constrained EM, MD-MD and HD-MD, in the first condition of the simulation study

Model	$\pi _K$		$\epsilon _{(i,j)}$, $i<j$		$\epsilon _{(i,j)}$, $i>j$	
	$\overline{{\sigma }}$	$\text {max}({\hat{\sigma }})$	$\overline{{\sigma }}$	$\text {max}({\hat{\sigma }})$	$\overline{{\sigma }}$	$\text {max}({\hat{\sigma }})$
Unconstrained EM	.0004	.0006	.0044	.0075	.0043	.0076
Constrained EM	.0007	.0009	.0068	.0084	.0068	.0084
HD-MD	.0004	.0006	.0038	.0061	.0037	.0062
MD-MD	.0004	.0006	.0039	.0070	.0037	.0064

By looking at the figure, it is clear that the only estimation method not obtaining biased estimates was the unconstrained maximum likelihood via the EM algorithm. Indeed, whenever monotonicity does not hold in the data, the constrained estimation is likely to be biased. Moreover, unconstrained estimation method was more efficient than the constrained version (smaller variance of the estimates, see Table 1).

The MD method obtained biased estimates with both the Hamming and the Manhattan distances. Comparing the parameter estimates obtained by the two, it is interesting to note that they produced very different parameter estimates. In the case of the Hamming distance (the two middle panels of Fig. 1), the bias seems systematic. In particular, for true parameter values that are in the middle of the interval (0, .05] no bias was observed; when true parameter values approach zero, over-estimations were observed; when true parameter values approach the maximum value of the interval, under-estimations were observed. This trend could suggest a sort of balancing between over- and under-estimations due to the restriction $\sum _{j \in L} \epsilon _{q}(i,j)=1$, for each level *i* of each item.

In the case of the Manhattan distance (the two bottom panels of Fig. 1), under-estimations were observed when the distance between the true and the observed levels was 3 or 2, that is, $\epsilon _{q}(3,1)$, $\epsilon _{q}(4,1)$, $\epsilon _{q}(4,2)$, $\epsilon _{q}(1,3)$, $\epsilon _{q}(1,4)$, $\epsilon _{q}(2,4)$, whereas over-estimations were observed when that distance was 1, that is, $\epsilon _{q}(4,3)$, $\epsilon _{q}(3,2)$, $\epsilon _{q}(2,1)$, $\epsilon _{q}(1,2)$, $\epsilon _{q}(2,3)$ and $\epsilon _q(3,4)$.

Concerning the recovery of the states probabilities $\pi _{K}$, the average absolute bias of the estimates was equal to $3.55 \times 10^{-5}$, $5.73 \times 10^{-4}$, $3.81 \times 10^{-5}$ and $4.81 \times 10^{-5}$, respectively, for the unconstrained and the constrained EM, the HD-MD and the MD-MD estimation procedures.

**Fig. 2:**
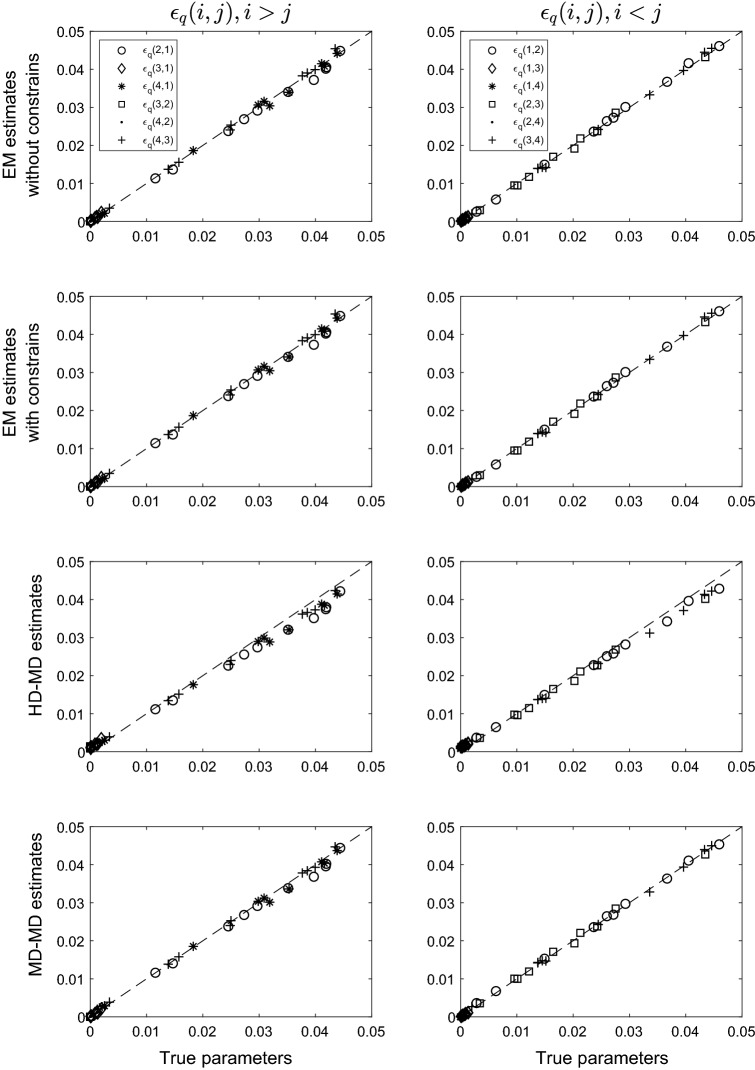
Parameter recovery of the PoLIM obtained by using the EM algorithm without constraints and with constraints, and by using the MD-HD and the MD-MD, when both the overall error condition and the monotonicity condition hold in the data

Figure 2 displays the results of the PoLIM’s $\epsilon _q(i,j)$ parameter recovery of the first condition (i.e., $\epsilon _{q}(i,i)=.95$) in the latter scenario, in which both the overall error and the monotonicity conditions held in the simulated data.

The figure reads as the previous one. The standard errors of the estimates are reported in Table 2.

**Table 2:** Average (columns 2, 4 and 6) and maximum (columns 3, 5 and 7) standard errors of the PoLIM’s parameter estimates obtained by the four estimation methods unconstrained EM, constrained EM, MD-MD and HD-MD, in the second condition

Model	$\pi _K$		$\epsilon _{(i,j)}$, $i<j$		$\epsilon _{(i,j)}$, $i>j$	
	$\overline{{\sigma }}$	$\text {max}({\hat{\sigma }})$	$\overline{{\sigma }}$	$\text {max}({\hat{\sigma }})$	$\overline{{\sigma }}$	$\text {max}({\hat{\sigma }})$
Unconstrained EM	.0007	.0009	.0039	.0076	.0037	.0069
Constrained EM	.0007	.0009	.0039	.0076	.0037	.0069
HD-MD	.0007	.0009	.0039	.0071	.0037	.0064
MD-MD	.0009	.0009	.0037	.0073	.0035	.0067

By looking at Fig. 2, it can be seen that, on the average, there are no differences between the constrained and unconstrained EM estimates, which are both unbiased. This result could suggest that the two estimation procedures perform equally well, in this condition. However, in the single sample this could not be true because there are no guarantees that the unconstrained parameter estimates satisfy monotonicity.

Surprisingly, the recovery of the parameters estimated by using the MD-MD method improved a lot. Indeed, the biases observed in the former scenario were considerably reduced. The same consideration does not apply to the HD-MD method, for which over- or under-estimations were obtained that were similar to those found in the first scenario (Fig. 1). A plausible explanation of this result could regard the type of the distance that is minimized in the two MD methods. Indeed, only the Manhattan distance respects the order on the response levels of *L*, as established in Eq. (7). For the same reason, as long as the MD-MD estimates have small bias when monotonicity holds true, they tend to have large bias when monotonicity does not hold. It is also interesting to note that the parameter estimates obtained by the MD-MD and by the HD-MD are more similar to one another when the monotonicity holds in the data than when it does not.

Concerning the estimates of the state parameters $\pi _{K}$ obtained by the three procedures, they were very close to the true values also in this scenario. The average absolute bias was in fact $3.57 \times 10^{-5}$, $5.47 \times 10^{-4}$, $3.63 \times 10^{-5}$ and $3.59 \times 10^{-5}$, respectively, for the constrained and unconstrained EM, the HD-MD and the MD-MD estimation procedures.

As regards conditions two and three of both scenarios, in which $\epsilon _{q}(i,i) \ge .85$ and $\epsilon _{q}(i,i) \ge .75$, respectively, similar results were obtained. The only difference was in the amount of the under- and over-estimations obtained when the estimation method of the PoLIM’s parameters was the HD-MD. As the amount of error in the data increased, the under- and the over-estimates also increased (the figures corresponding to the results of these two conditions can be found in the supplementary material of the paper).

## Empirical Application

In this study, the parameters of the PoLIM with and without monotonicity constraints were estimated on a real data set by using the corresponding maximum likelihood procedures. Moreover, the model was estimated also by minimum discrepancy by using both the Hamming and the Manhattan distances. In the case of the MD estimation procedure, only the unconstrained model was estimated. The likelihoods and the parameter estimates of the four models were compared.

### The Data Set

The data were composed of the responses of $N=3,673$ individuals to the Italian version (Pedrabissi and Santinello 1989; Vidotto and Bertolotti 1991) of the reduced form of the State Trait Anxiety Inventory form Y-1 (STAI-Y-1; Spielberger 1983). It is a psychological self-report questionnaire investigating the “state” anxiety. The STAI Y-1R consists of 10 items for which the subject establishes his agreement on a 4-point Likert scale, from “not at all” (coded as 0) to “very much” (coded as 3). Six items of the test have a positive wording (i.e., the higher the level, the higher the state anxiety), whereas the others have a negative wording (i.e., the higher the level, the lower the state anxiety). The responses to the latter items were re-scored prior to analyses. All participants signed the informed consent and were asked to answer to all the items of the questionnaire. No time limit was imposed.

### Generation of the Belief Structure and Methods

For generating the structure, the data-driven extraction procedure recently proposed by de Chiusole et al. (2019) has been used. It is an adaptation of the *k*-median clustering algorithm to KST, when data consist in answers to items having more than two response categories. Thus, it can be used for extracting structures from polytomous data.

This *k*-median algorithm is an extension of the well-known *k*-means algorithm to ordinal data. It consists of an iteration of two steps: the “pattern classification step” and the “centroid adjustment step.” The former consists of partitioning the whole set of the observed patterns into the classes represented by centroids (states) that minimize the intraclass dissimilarity based on the Manhattan distance. The latter step consists in updating the centroids in order to minimize the Manhattan discrepancy from the data by using the median.

The algorithm requires a set $\mathcal {I}$ of fixed cardinality, containing the initial centroids. These centroids can be randomly extracted out of the observed response patterns.

The whole data sample ${\mathcal {D}}$ was randomly partitioned into three sets: The first set ${\mathcal {D}}_1$ of 1,782 subjects was used for extracting a given number of structures; the second set ${\mathcal {D}}_2$ of 891 subjects was used for selecting the best extracted structure; and the third set ${\mathcal {D}}_0$ of 1,000 subjects was used in the end, for testing the “best” extracted structure. The sizes of the two sets ${\mathcal {D}}_1$ and ${\mathcal {D}}_2$ were obtained by subtracting 1,000 from the size of ${\mathcal {D}}$ and by dividing the resulting number into about two thirds for ${\mathcal {D}}_1$ and one third for ${\mathcal {D}}_2$.

A number of 100 different random partitions have been generated from ${\mathcal {D}} \setminus {\mathcal {D}}_0$. For each of them, sixteen different cardinalities for the set ${\mathcal {I}}$ were considered, which varied in the interval $\{50, 100, . . . , 800\}$ with a step of 50. Thus, $100 \times 16=$ 1,600 different structures have been extracted. The best one was selected by using the minimax criterion introduced by de Chiusole et al. (2019) and described below.

The minimax criterion here considered is a discrepancy index computed on the two non-symmetric discrepancies $\Delta ({\mathcal {D}}_2,\mathcal {K})$ and $\Delta (\mathcal {K},{\mathcal {D}}_2)$. They allow for computing the average “distance” between the validation set ${\mathcal {D}}_2$ and each of the extracted structures $\mathcal {K}$. The former discrepancy is computed by

$$\begin{aligned} \Delta ({\mathcal {D}}_2,\mathcal {K})=\frac{1}{|{\mathcal {D}}_2|}\sum _{X \in {\mathcal {D}}_2} \min \{d_M(X,K) : K \in \mathcal {K}\}, \end{aligned}$$

where $d_M(X,K)$ is the Manhattan discrepancy between response pattern *X* and state *K*, computed as in Eq. (18) in Appendix, whereas the latter is computed by

$$\begin{aligned} \Delta (\mathcal {K},{\mathcal {D}}_2) = \frac{1}{|\mathcal {K}|}\sum _{K \in \mathcal {K}} \min \{d_M(X,K) : X \in {\mathcal {D}}_2\}. \end{aligned}$$

Let ${\mathbf {K}}$ be the collection of all the knowledge structures extracted by *k*-median. Let, moreover,

$$\begin{aligned} z=\underset{\mathcal {K}\in {\mathbf {K}}}{\min } \;\; \max \{\Delta (\mathcal {K},{\mathcal {D}}_2),\Delta ({\mathcal {D}}_2,\mathcal {K})\} \end{aligned}$$

be the minimum value across all the extracted structures of the maximum between the two discrepancies $\Delta (\mathcal {K},{\mathcal {D}}_2),\Delta ({\mathcal {D}}_2,\mathcal {K})$. In de Chiusole et al. (2019), it is proved that the structure $\hat{{\mathcal {K}}} \in {\mathbf {K}}$ for which $\max \{\Delta (\mathcal {K},{\mathcal {D}}_2),\Delta ({\mathcal {D}}_2,\mathcal {K})\} = z$ is the “best” in the sense that for any other structure one of the two discrepancies $\Delta (\mathcal {K},{\mathcal {D}}_2),\Delta ({\mathcal {D}}_2,\mathcal {K})$ is higher than *z*.

The four versions of the PoLIM based on this “best” selected structure were fitted to ${\mathcal {D}}_0$. Concerning the two models obtained by the EM estimation procedures, it is not trivial to compare them because one is an unconstrained model and the other one is constrained. In this specific case, they have the same number of free parameters but different parameter spaces. Nonetheless, the two versions of the model were compared with respect to their log-likelihoods. The same was done for the PoLIM versions based on the two MD estimation procedures.

### Results and Discussion

The procedure used for extracting the structure from the data selected a structure containing 148 states. Surprisingly, the extracted structure was quite small, compared to the $4^{10} =1,048,576$ potential states (and response patterns). This may be due to the sample size of the data set ${\mathcal {D}}_1$ used for extracting the structure (i.e., 1,782 with 842 distinct response patterns), which is also somewhat limited compared to the large number of possible response patterns. Nevertheless, this particular structure represents the best trade-off between “fit” and “complexity” provided by the minimax criterion.

The PoLIM parameters were estimated by using the constrained and the unconstrained EM algorithms, and by minimum discrepancy based on both the Hamming and the Manhattan distances. The empirical test of the model identifiability described in Sect. 4.1 did not detect any kind of problem. In fact, the maximal standard deviation of the parameter estimates obtained by repeatedly estimating them on the same sample from different starting points was $3.17 \times 10^{-5}$.

The log-likelihoods of the two PoLIMs estimated with the EM were 7,329 and 7,336, respectively, for the unconstrained and the constrained versions. The log-likelihoods of the two PoLIMs estimated with the MD were 7,646 and 7,597, respectively, when the Hamming and the Manhattan distances were used. The following considerations can be drawn: (1) The two EM estimation procedures obtained smaller log-likelihoods than the two MD procedures; (2) Comparing the two MD procedures, the one based on the Manhattan distance obtained the smallest log-likelihood; (3) Comparing the two EM procedures, the unconstrained PoLIM exhibited the smallest log-likelihood, even if by a small amount. On the whole, these results could suggest that for the most part of the items the monotonicity constraint holds in the data, with few exceptions. For verifying this, the unconstrained parameter estimates were analyzed (the estimates of the constrained PoLIM are provided as supplementary material of the paper).

**Fig. 3:**
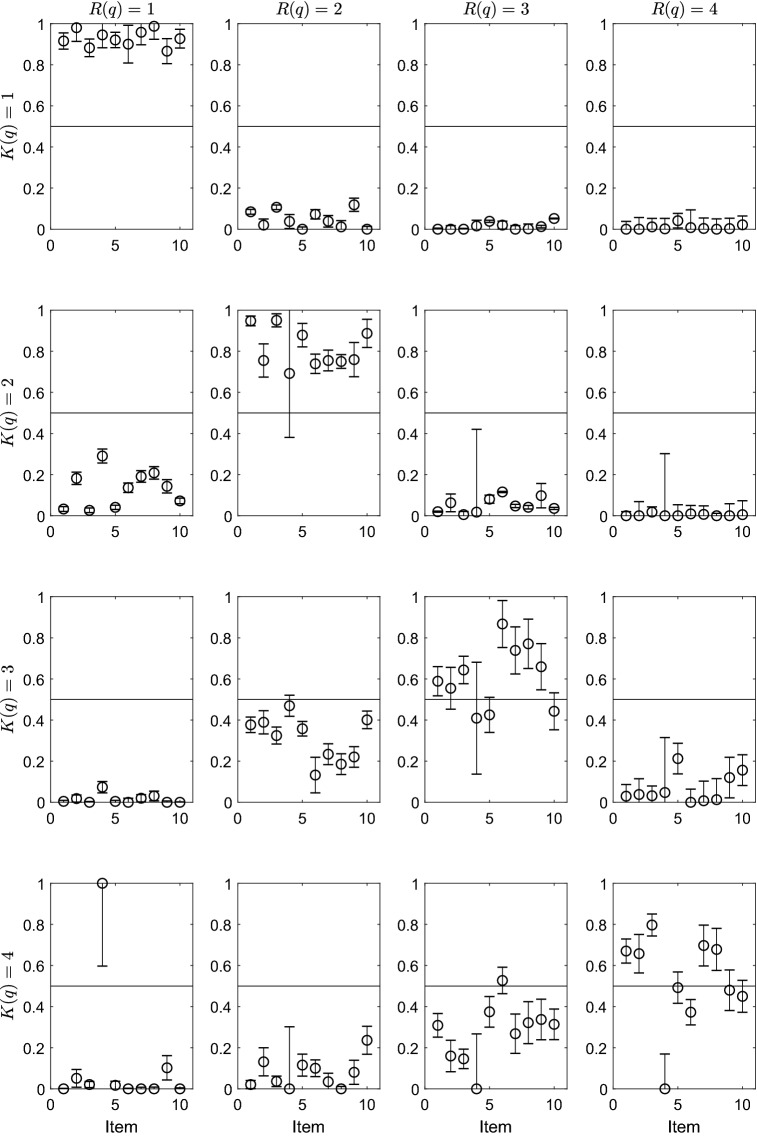
Parameter estimates of the PoLIM obtained by using the unconstrained EM on real data. The error bars represent the bootstrapped standard errors of the estimates

Figure 3 displays the unconstrained PoLIM’s parameter estimates obtained for each item $q \in Q$ (*x*-axis of the panels). Each panel of the figure displays the estimates of a particular $\epsilon _{q}(i,j)$. Rows of the figure represent the *K*(*q*) values, whereas columns represent the *R*(*q*) values. The error bars represent the bootstrapped standard errors of the estimates.

Several interesting results have been found. In most cases, the highest estimates were observed for the ${\hat{\epsilon }}_{q}(i,i)$ parameters (diagonal panels), with the exception of ${\hat{\epsilon }}_{4}(3,3)$, which was smaller than ${\hat{\epsilon }}_{4}(3,2)$, and of ${\hat{\epsilon }}_{6}(4,4)$, which was smaller than ${\hat{\epsilon }}_{6}(4,3)$. This result suggests that the modality condition is respected by the ${\hat{\epsilon }}_q{(i,j)}$ parameters for almost all the levels of almost all the items.

Concerning the other $\epsilon _{q}(i,j)$ parameters, moving away from the diagonal values, an overall decrease in the estimates was observed. This result could suggest that a monotonicity condition across the $\epsilon _{q}(i,j)$ parameters is plausible. Nonetheless, some parameter estimates did not satisfy the monotonicity (e.g., ${\hat{\epsilon }}_{6}(4,3)> {\hat{\epsilon }}_{6}(4,4)> {\hat{\epsilon }}_{6}(4,2)> {\hat{\epsilon }}_{6}(4,1)$).

A separate comment has to be done on Item 4. It is rather evident that the standard errors of the estimates of parameters ${\hat{\epsilon }}_{4}(2,2)$, ${\hat{\epsilon }}_{4}(2,3)$, ${\hat{\epsilon }}_{4}(2,4)$, ${\hat{\epsilon }}_{4}(3,3)$, ${\hat{\epsilon }}_{4}(3,4)$, ${\hat{\epsilon }}_{4}(4,1)$, ${\hat{\epsilon }}_{4}(4,2)$, ${\hat{\epsilon }}_{4}(4,3)$, ${\hat{\epsilon }}_{4}(4,4)$ of Item 4 are quite large. A possible explanation could be a poor information in the data concerning some levels of Item 4 (only 7 subjects out of 1,000 chose level 4 for this item).

**Fig. 4:**
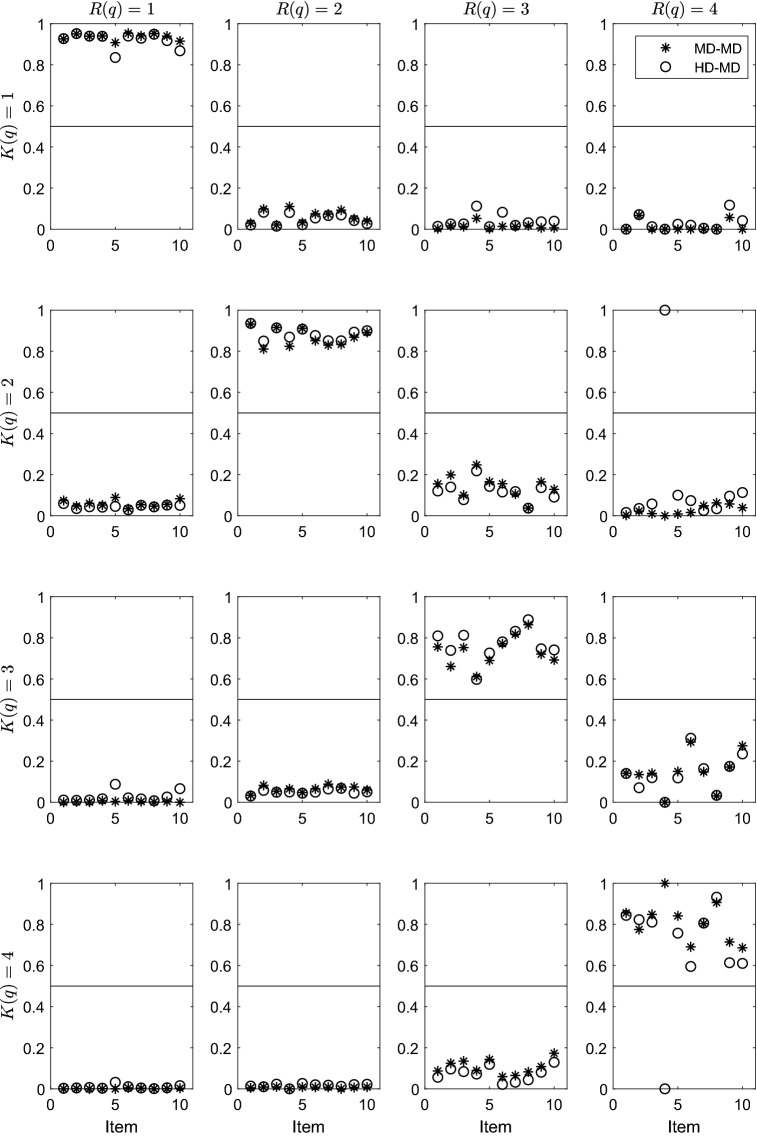
Parameter estimates of the PoLIM estimated via the MD on a real data set. Stars in the panels refers to the estimates obtained by using the Manhattan distance, whereas circles refers to the estimates obtained by using the Hamming distance

Figure 4 displays the parameter estimates obtained by the MD estimation procedure applied by using the Hamming (circles in the panels) and the Manhattan (stars in the panels) distances. It is worth noticing that standard errors of the estimates are not given here. This is because, at the best of our knowledge, there is not yet a way for obtaining them for the parameters estimated by MD.

The interesting result here is that the MD-MD estimates are quite similar to the ones obtained by the HD-MD. In the simulations, this happens when the monotonicity condition holds in the data. This provides further evidence supporting the conjecture that the monotonicity condition is respected by the data.

## Conclusion

Building upon the polytomous extension of KST proposed in the literature (Schrepp 1997; Stefanutti et al. 2020), the present work defined a probabilistic framework over it. A version of the BLIM for polytomous data has been proposed that is called PoLIM. Similar to the BLIM, the PoLIM considers a probability distribution over the states of the structure (which accounts for the different frequency of the states in the population) and error probabilities for the items (which account for inconsistencies between “true” and observed item levels). At least two relevant differences between the BLIM and the PoLIM can be outlined. The first one concerns the number of error probabilities that are needed to account for the aforementioned inconsistencies. While two error probabilities per item are sufficient in the BLIM, a larger number is required in the PoLIM. This is because the number of ways in which “true” and observed item levels can differ increases with the number of levels in *L*. The second difference between the two models concerns the ways in which monotonicity of the error probabilities can be defined. There are only two distinct forms of monotonicity for the error probabilities of the BLIM that are named row and column monotonicity in this article. In the PoLIM, each of these two forms of monotonicity can be generalized in at least three different ways, which have been denoted as modality, monotonicity and overall error.

Two different types of algorithms for estimating the PoLIM’s parameters have been derived. The former type is the well-known maximum likelihood estimation via the EM algorithm. More in detail, two different variants of the EM have been derived, one imposing monotonicity constraints across the error parameters of the items and one without any kind of constraints. The latter type of algorithm developed for estimating the PoLIM is by minimum discrepancy.

All the algorithms have been tested in a simulation study. The algorithms differed in their capability of recovering the true parameter values. The unconstrained EM estimates of the error probabilities largely resembled the true values, whether (row) monotonicity was satisfied or not. Instead, the constrained EM produced unbiased estimates only when the (row) monotonicity was satisfied by the data. Some rather small biases were observed in HD-MD estimates whose size was not affected by monotonicity. Conversely, the MD-MD estimates largely reproduced the true values only when monotonicity was satisfied.

An application of the PoLIM to empirical data showed that the model can be successfully applied to polytomous data from psychological assessment, paving the way to a number of applications of KST outside the area of knowledge and learning assessment.

As for the most part of latent class models, the identifiability of the parameters of the BLIM is an important issue. In general, the BLIM is not globally identifiable. Nonetheless, it proved to be locally identifiable under rather standard conditions. In the last years, the identifiability of the BLIM has been widely explored e.g., Heller 2017; Spoto et al. 2012, 2013; Stefanutti et al. 2012, 2018; Stefanutti and Spoto 2020 providing a more in-depth understanding of the characteristics of the unidentifiable dichotomous structures and providing useful tools for testing the identifiability of the model. For what concerns the PoLIM, this issue has not been studied in detail yet. Possible ways for detecting PoLIM’s unidentifiability, other than its empirical evaluation conducted in the present research, may refer either to the classical analysis of the rank of the Jacobian matrix, or to a tentative extension to the polytomous case of the transformational approach developed for the dichotomous one (Spoto et al. 2012, 2013; Stefanutti et al. 2018; Stefanutti and Spoto 2020). For the dichotomous case, some solutions have been provided to cope with the unidentifiability issue, although not completely effective. The most promising one was the introduction of equivalent items, that is, items that are contained in all the same states in a structure (Spoto et al. 2013). This operation proved to be effective in most cases, but Heller (2017) showed that it fails to solve unidentifiability issues under particular conditions. The analytical study of the identifiability of PoLIM needs to be carried out, especially with respect to local identifiability, while it should be reasonable to assume that it is not globally identifiable.

One of the most relevant applications of KST is adaptive assessment. It aims at uncovering the state of a student by presenting her with only a minimal number of items. Adaptive assessment has been found to be a better experience for individuals because the test is tailored on each of them instead of being fixed (Deville 1993; Linacre 2000). The probabilistic framework introduced in this article could form the basis for the development of a polytomous version of the adaptive assessment procedure proposed in KST by Falmagne and Doignon (1988a). This procedure has been studied by Anselmi et al. (2016), Heller and Repitsch (2012), and Hockemeyer (2002), and applied to psychological assessment by Donadello et al. (2017). The procedure is currently used by the ALEKS (acronym for Assessment and LEarning in Knowledge Spaces) system to adaptively assessing students’ knowledge in mathematics, business, science and behavioral science (see, e.g., Reddy an Harper 2013; Falmagne and Doignon 2011) . Such a procedure would require the introduction of a likelihood function over the structure that expresses the plausibility of the states. The likelihood function is updated at each step of the assessment, according to an individual’s response to the presented item and to the error probabilities of the PoLIM. The assessment would stop when a large enough portion of the likelihood is concentrated on a unique state, which then is regarded as the uncovered state of the individual.

### Electronic supplementary material

Below is the link to the electronic supplementary material.

Supplementary material 1 (pdf 395 KB)

## Estimating the Parameters of the PoLIM

### Estimation by Unconstrained Maximum Likelihood

Maximum likelihood estimation of the parameters of the BLIM model via the expectation–maximization (EM) algorithm was developed by Stefanutti and Robusto (2009). In this section, an EM algorithm is described which can be applied for estimating the parameters of the PoLIM.

For both *Q* and *L* nonempty and finite, the data set is denoted by the pair $(L^Q,F)$, where $F:L^Q \rightarrow \mathbb {R}^+$ is a frequency distribution over the response patterns $R \in L^Q$. Thus, $F(R) \ge 0$ is the observed frequency of response pattern $R \in L^Q$. The sample size is $N = \sum _{R \in L^Q}F(R)$.

For $i=1,2,\ldots ,N$, let $R_i \in L^Q$ denote the response pattern of the *i*-th subject in the data set. Let $K_i \in \mathcal {K}$ be the (unobservable) state of this subject. The “complete log-likelihood” of the PoLIM model is

$$\begin{aligned} L&= \sum _{i=1}^N \ln P(R_i|K_i)\pi _{K_i} \\&= \sum _{i=1}^N \ln \left( \prod _{q \in Q} \epsilon _q(K_i(q),R_i(q))\right) + \sum _{i=1}^N \ln \pi _{K_i}\\&= \sum _{i=1}^N \sum _{q \in Q} \ln \epsilon _q(K_i(q),R_i(q)) + \sum _{i=1}^N \ln \pi _{K_i}. \end{aligned}$$

The conditional expectation of the complete log-likelihood, given the vector $\theta$ of the model parameters, and the data set $(L^Q,F)$ is

$$\begin{aligned} \mathcal {E}=&E\left( \sum _{i=1}^N \sum _{q \in Q} \ln \epsilon _q(K_i(q),R_i(q)) + \sum _{i=1}^N \ln \pi _{K_i} | \theta \right) \\&=\sum _{i=1}^N \sum _{q \in Q} E\left( \ln \epsilon _q(K_i(q),R_i(q))|\theta \right) + \sum _{i=1}^N E\left( \ln \pi _{K_i} | \theta \right) . \end{aligned}$$

Concerning the left-hand term of the sum:

$$\begin{aligned} \mathcal {E}_1&= \sum _{i=1}^N \sum _{q \in Q} E\left( \ln \epsilon _q(K_i(q),R_i(q))|\theta \right) \\&= \sum _{i=1}^N \sum _{q \in Q} \sum _{K \in \mathcal {K}} P_\theta (K|R_i) \ln \epsilon _q(K_i(q),R_i(q)) \\&= \sum _{q \in Q}\sum _{R \in L^Q} \sum _{K \in \mathcal {K}} F(R)P_\theta (K|R_i) \ln \epsilon _q(K_i(q),R_i(q)), \end{aligned}$$

where

$$\begin{aligned} P_\theta (K|R_i) = \frac{P_\theta (R_i|K)\pi _K}{\sum _{K' \in \mathcal {K}} P_\theta (R_i|K')\pi _{K'}} \end{aligned}$$

is the posterior probability of *K*, given response pattern $R_i$, obtained by an application of the Bayes theorem with model parameters $\theta$.

As for the right-hand term,

$$\begin{aligned} \mathcal {E}_2&= \sum _{i=1}^N E\left( \ln \pi _{K_i} | \theta \right) \\&= \sum _{R \in L^Q}\sum _{K \in \mathcal {K}} F(R)P_{\theta }(K|R)\ln \pi _K. \end{aligned}$$

This completes the so-called expectation step of the EM. The “maximization step” consists of finding model parameter values that maximize the negative of (thus, indeed, minimize) the two quantities $\mathcal {E}_1$ and $\mathcal {E}_2$. It is important to observe that $\mathcal {E}_1$ only depends on the $\epsilon _q$ parameters, whereas $\mathcal {E}_2$ only depends on the $\pi _K$ parameters. For both quantities, minimization is constrained. For $\mathcal {E}_1$, the constraint is given by the fact that

13 $$\begin{aligned} \sum _{j \in L} \epsilon _q(i,j) = 1 \end{aligned}$$

for all $i \in L$ and all $q \in Q$. For $\mathcal {E}_2$, the constraint is

14 $$\begin{aligned} \sum _{K \in \mathcal {K}} \pi _K = 1. \end{aligned}$$

In both cases, Lagrange multipliers can be applied. Given the constraints, the quantity that has to be maximized by the $\epsilon _q$ parameters is thus

$$\begin{aligned} \mathcal {E}_1^*&= \mathcal {E}_1 + \sum _{q \in Q}\sum _{i \in L} \lambda _{qi} \left( 1 - \sum _{j \in L}\epsilon _q(i,j)\right) , \\&= \sum _{q \in Q}\sum _{R \in L^Q} \sum _{K \in \mathcal {K}} F(R)P_\theta (K|R) \ln \epsilon _q(K(q),R(q)) + \sum _{q \in Q}\sum _{i \in L} \lambda _{qi} \left( 1 - \sum _{j \in L}\epsilon _q(i,j)\right) , \end{aligned}$$

where $\lambda _{qi}$ is the Lagrange multiplier for level *i* of item *q*. Differentiating with respect to $\epsilon _u(v,z)$, $u \in Q$, $v,z \in L$,

$$\begin{aligned} \frac{\partial \mathcal {E}_1^*}{\partial \epsilon _u(v,z)} = \frac{1}{\epsilon _u(v,z)}\sum _{\mathcal {K}_{uv}}\sum _{\mathcal {R}_{uz}} F(R)P_\theta (K|R)-\lambda _{uv}. \end{aligned}$$

By setting to zero this derivative and solving for $\epsilon _u(v,z)$, one obtains

15 $$\begin{aligned} \epsilon _u(v,z) = \frac{1}{\lambda _{uv}}\sum _{\mathcal {K}_{uv}}\sum _{\mathcal {R}_{uz}} F(R)P_\theta (K|R). \end{aligned}$$

Summing both sides of the equation across all $z \in L$,

$$\begin{aligned} \sum _{z \in L}\epsilon _u(v,z) = \frac{1}{\lambda _{uv}}\sum _{z \in L}\sum _{\mathcal {K}_{uv}}\sum _{\mathcal {R}_{uz}} F(R)P_\theta (K|R). \end{aligned}$$

By the constraint (13), $\sum _{z \in L}\epsilon _u(v,z)=1$, hence

$$\begin{aligned} \lambda _{uv} = \sum _{z \in L}\sum _{\mathcal {K}_{uv}}\sum _{\mathcal {R}_{uz}} F(R)P_\theta (K|R). \end{aligned}$$

By inserting the right-hand side of this last equation into (15), one finally obtains the re-estimation equation of the $\epsilon _q(i,j)$ parameters by the EM algorithm:

$$\begin{aligned} \epsilon _u(v,z) = \frac{\sum _{\mathcal {K}_{uv}}\sum _{\mathcal {R}_{uz}} F(R)P_\theta (K|R)}{\sum _{z \in L}\sum _{\mathcal {K}_{uv}}\sum _{\mathcal {R}_{uz}} F(R)P_\theta (K|R)}. \end{aligned}$$

The re-estimation equation of the $\pi _K$ probabilities is identical to those for the dichotomous BLIM, described in, e.g., Stefanutti and Robusto (2009).

### Estimation by Constrained Maximum Likelihood

The maximum likelihood estimation of the parameter of the PoLIM with monotonicity constraints can be obtained via the EM algorithm in a rather straightforward way. The reparameterization described in Sect. 3.2 is a bijective transformation of the conditional probabilities $\epsilon _q$ into the odds $\upsilon _q$ and $\omega _q$. The monotonicity constraints are respected if both these odds lie with the (0, 1) open interval for each of the items *q* and every pair $i,j \in L$ of levels.

The EM algorithm described in the previous section for the unconstrained model can be modified as follows. At the outset, the initial guesses of the $\upsilon _q$ and $\omega _q$ parameters are randomly generated so that all of them lie within the (0, 1) open interval. The initial guesses of the $\epsilon _q$ probabilities are obtained from the $\upsilon _q$ and $\omega _q$ via the proper transformation (Sect. 3.2). Then, at each iteration $n>0$ of the EM algorithm, the $\epsilon _q^{(n+1)}$ parameters are obtained by applying the unconstrained updating formulae of the EM. They are then transformed into the $\omega _q^{(n+1)}$ and $\upsilon _q^{(n+1)}$ odds. For each item $q \in Q$, and pair of levels $i,j \in L$ such that $i < j$ the condition $\upsilon _{qij}^{(n+1)} \in (0,1)$ is tested. If the test is successful, then nothing need be done; if the test fails (in this case one has $\upsilon _{qij}^{(n+1)}>1$), then $\upsilon _{qij}^{(n+1)}$ is set to a value in between $\upsilon _{qij}^{(n)}$ and 1 (different criteria can be applied for choosing such value; randoms generation with continuous uniform distribution in the interval $[\upsilon _{qij}^{(n)},1]$ was chosen in the implemented procedure). A similar procedure is followed for the $\omega _q$ parameter updates. After all checks are accomplished, the $\upsilon _q^{(n+1)}$ and $\omega _q^{(n+1)}$ parameters are transformed back to the $\epsilon _q^{(n+1)}$ parameters, and if a termination criterion is not reached, a new iteration of the EM algorithm takes place.

### Estimation by Minimum Discrepancy

This section presents an extension of the minimum discrepancy (MD) method (Heller and Wickelmaier 2013) for estimating the parameters of the PoLIM. Assumptions of the MD method are:

1. The response pattern is always generated by a belief state that is at a minimum distance from it.
2. The belief states at a minimum distance from the response pattern have the same probability of generating it.

The data set is denoted via a function $F: L^Q \rightarrow \mathbb {R}^+$ assigning observed frequencies to elements in $L^Q$ and a collection $\mathcal {R}= \{R \in L^Q: F(R) > 0\}$ of observed elements in $L^Q$, which are called *response patterns*.

Let $d:L^Q \times L^Q \rightarrow \mathbb {R}^+ \cup \{0\}$ be a metric. The *distance* of a response pattern $R \in L^Q$ from the structure $\mathcal {K}$, based on the metric *d*, is defined to be

16 $$\begin{aligned} D(R,\mathcal {K}) = \min \{d(R,K):K \in \mathcal {K}\}. \end{aligned}$$

Among possible metrics *d*, the Hamming and the Manhattan distances are considered here. The Hamming distance between $R \in L^Q$ and $K \in \mathcal {K}$ is defined as

17 $$\begin{aligned} d_H(R,K) = \sum _{q \in Q} \delta _H(R(q),K(q)), \end{aligned}$$

where $\delta _H$ is given in Eq. (5). The Manhattan distance between $R \in L^Q$ and $K \in \mathcal {K}$ is defined as

18 $$\begin{aligned} d_M(R,K) = \sum _{q \in Q} \delta _M(R(q),K(q)), \end{aligned}$$

where $\delta _M$ is defined in Eq. (6).

Under Assumption A1, let $z_{RK}$ be an indicator function obtained by defining, for all $R \in \mathcal {R}$ and all $K \in \mathcal {K}$

$$\begin{aligned} z_{RK} = {\left\{ \begin{array}{ll} 1 &{} \text {if } d(R,K) = D(R,\mathcal {K}), \\ 0 &{} \text {otherwise}. \\ \end{array}\right. } \end{aligned}$$

For a given response pattern $R \in \mathcal {R}$, let $z_{R+} = \sum _{K \in \mathcal {K}} z_{RK}$ denote the number of belief states in $\mathcal {K}$ that are at a minimum distance from *R*. Under Assumption A2, the estimate of the conditional probability of a belief state $K \in \mathcal {K}$, given a response pattern $R \in \mathcal {R}$, is $P(K|R) = z_{RK}/z_{R+}$.

The MD estimators are derived in the following way. The probability distribution over the belief states $K \in \mathcal {K}$ is estimated by

$$\begin{aligned} {\hat{\pi }}_K = \frac{\sum _{R \in \mathcal {R}} (z_{RK}/z_{R+}) F(R)}{\sum _{R \in \mathcal {R}}F(R)}. \end{aligned}$$

With $i, j \in L$ being any two levels, the conditional probability of observing response $R(q) = j$ to item *q*, given the level $K(q) = i$ assigned by the belief state *K* to *q*, is estimated by

$$\begin{aligned} {\hat{\epsilon }}_{q}(i,j) = \frac{\sum _{K \in \mathcal {K}_q(i)}\sum _{R \in \mathcal {R}_q(j)}(z_{RK}/z_{R+})F(R)}{\sum _{K \in \mathcal {K}_q(i)}\sum _{R \in \mathcal {R}}(z_{RK}/z_{R+})F(R)}, \end{aligned}$$

where $\mathcal {K}_q(i)$ is the collection of belief states in $\mathcal {K}$ assigning level *i* to item *q* and $\mathcal {R}_q(j)$ is the collection of response patterns in $\mathcal {R}$ assigning level *j* to item *q*. Unlike the EM algorithm, the MD method calculates ${\hat{\pi }}_K$ and ${\hat{\epsilon }}_{q}(i,j)$ in a non-iterative way. This makes it particularly appealing for its computational efficiency.
