# Supplementary material for: Extending the Basic Local Independence Model to Polytomous Data
Source: Psychometrika. 2020 Sep 21;85(3):684–715. doi: 10.1007/s11336-020-09722-5 (PMC7599199; doi:10.1007/s11336-020-09722-5)
Supplement: Supplementary file 1 — Supplementary material 1 (pdf 395 KB) [file 11336_2020_9722_MOESM1_ESM.pdf]

Web Supplementary Material of the article “Extending the basic local independence model to polytomous data”

A list of figures follows. Each figure displays the results obtained in a different condition of the simulation study (see Section 4.1 of the article for a detailed description of the simulation design) for the two scenarios “Monotonicity constraints do not hold in the data” (Figures 1 to 5) and “Monotonicity constraints hold in the data” (Figures 6 to 10). Furthermore, Figure 11 shows the parameter estimates of the constrained PoLIM obtained in the empirical application.

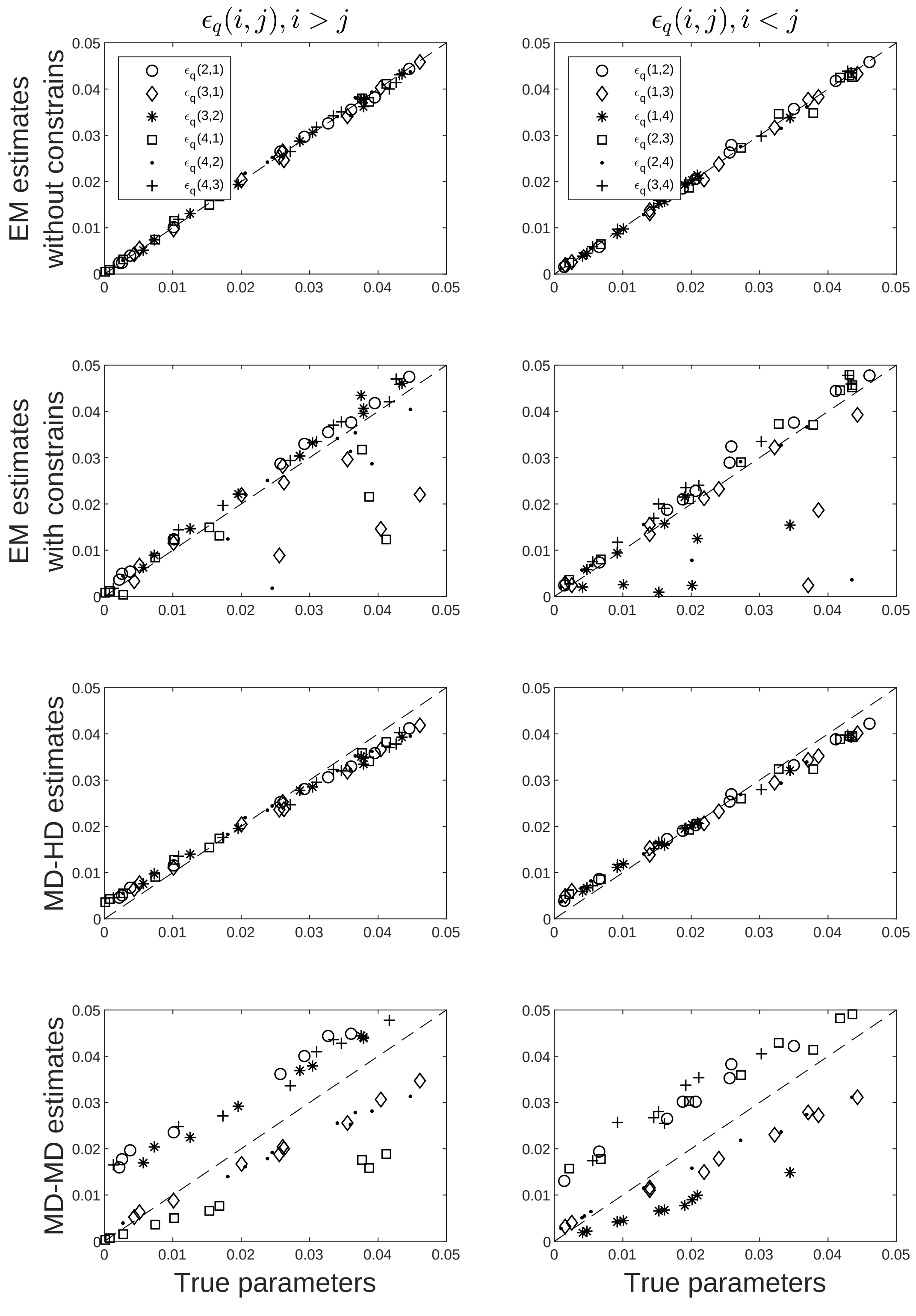

Figure 1: Parameter recovery of the PoLIM obtained by the four estimation procedures when monotonicity constraints do not hold in the data, sample size is $N = 2,000$ and $\epsilon_q i, i \geq .85$.

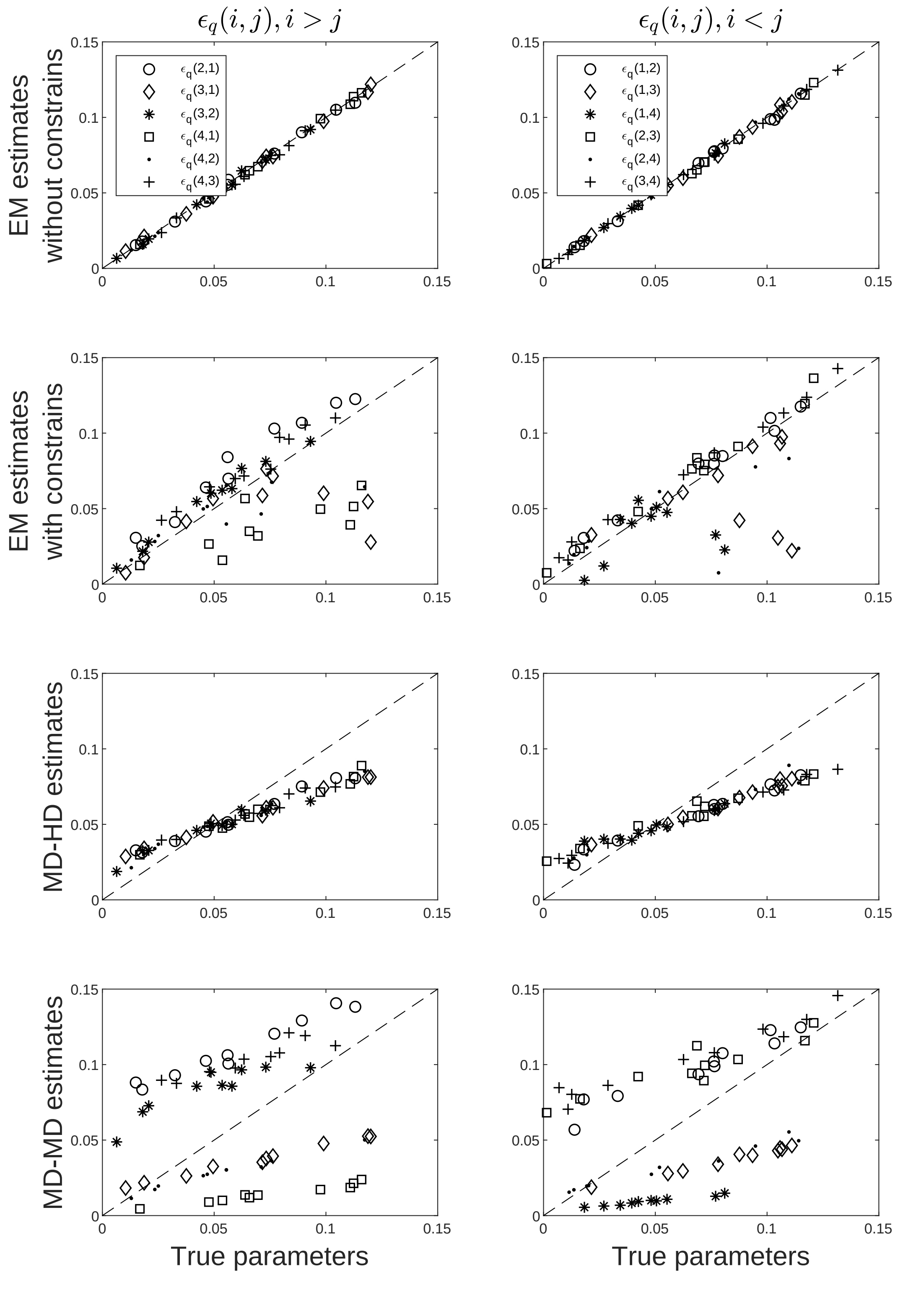

Figure 2: Parameter recovery of the PoLIM obtained by the four estimation procedures when monotonicity constraints do not hold in the data, sample size is $N = 2,000$ and $\epsilon_q i, i \geq .75$.

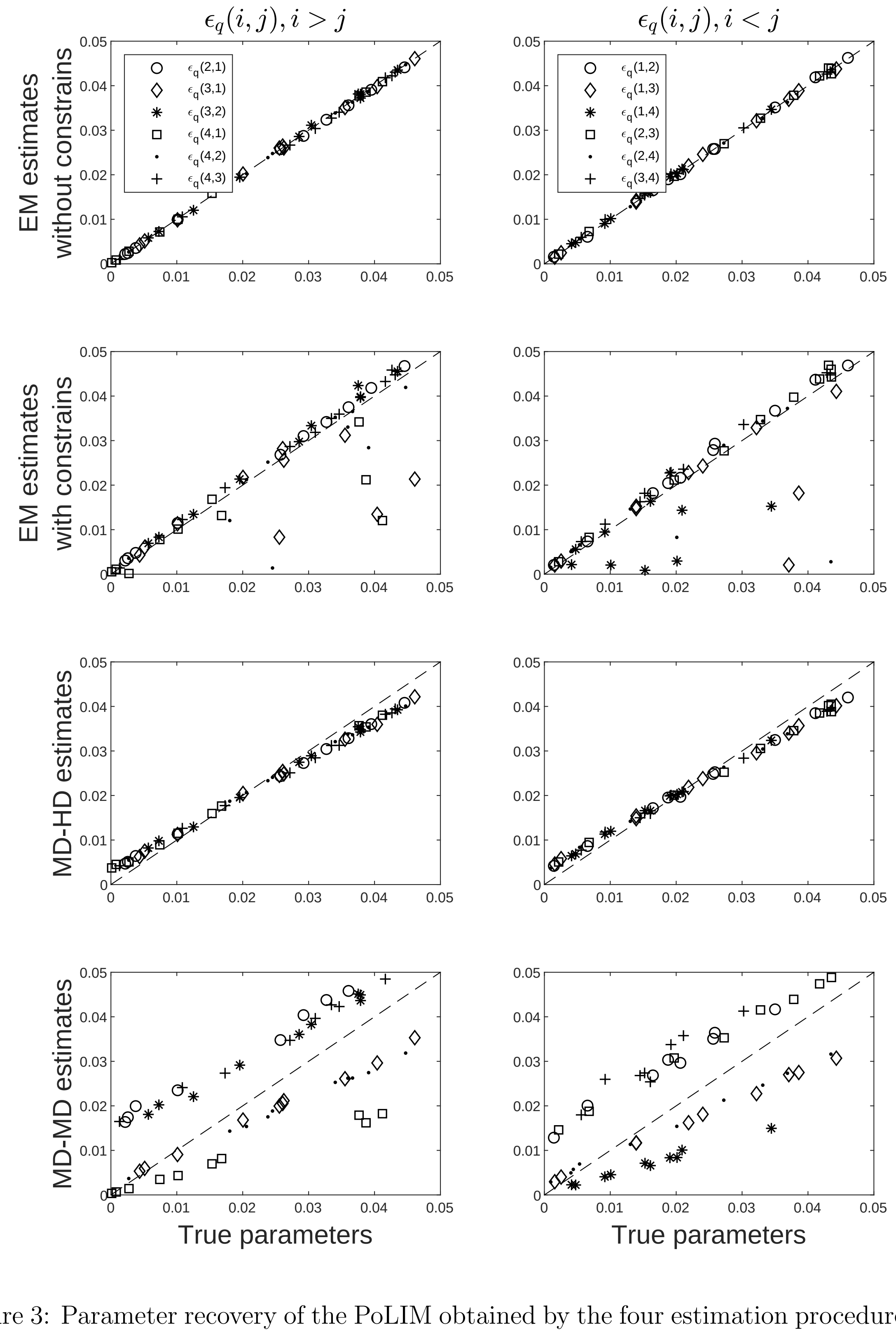

Figure 3: Parameter recovery of the PoLIM obtained by the four estimation procedures when monotonicity constraints do not hold in the data, sample size is $N = 5,000$ and $\epsilon_q i, i \geq .95$.

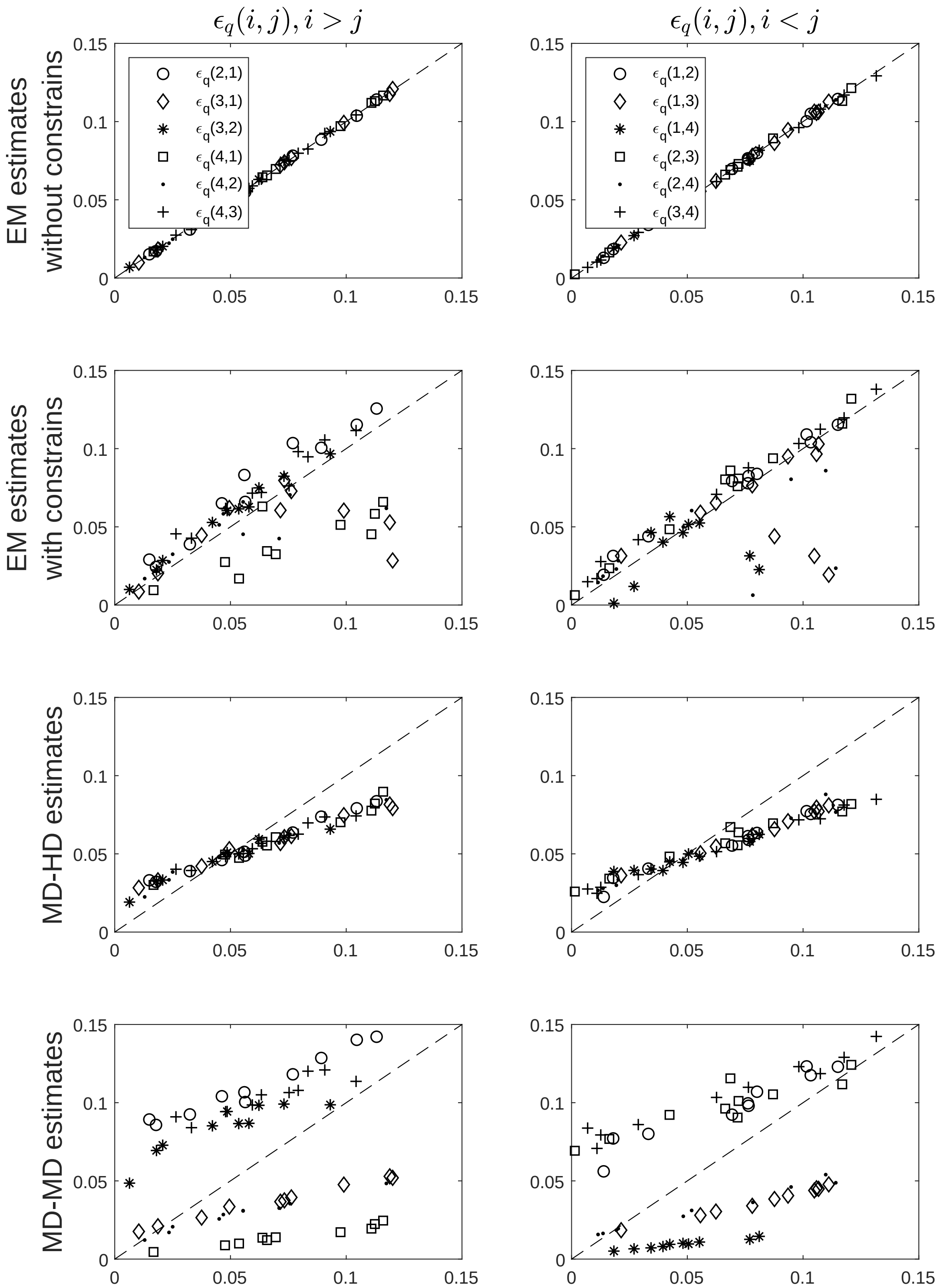

Figure 4: Parameter recovery of the PoLIM obtained by the four estimation procedures when monotonicity constraints do not hold in the data, sample size is $N = 5,000$ and $\epsilon_q i, i \geq .85$.

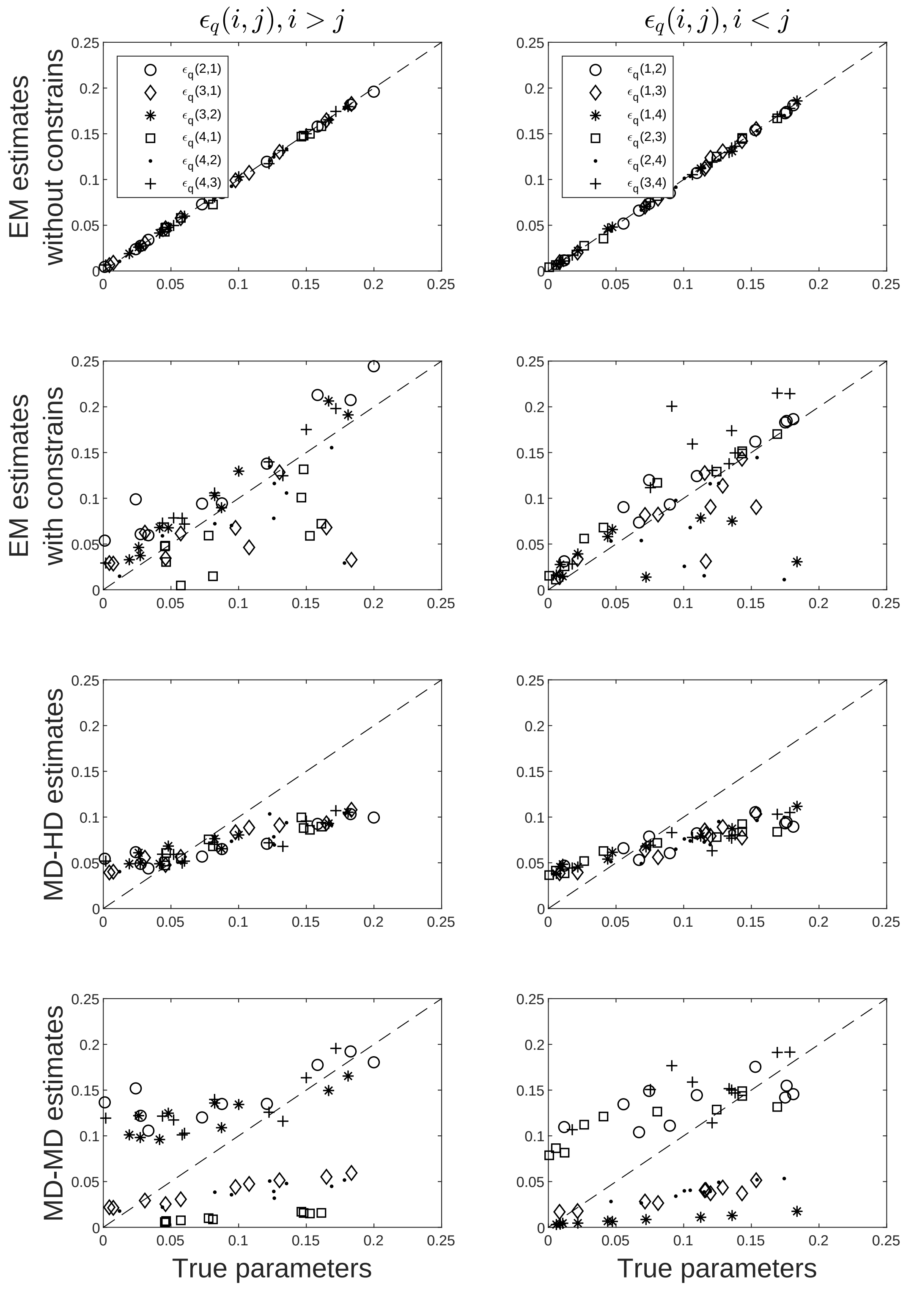

Figure 5: Parameter recovery of the PoLIM obtained by the four estimation procedures when monotonicity constraints do not hold in the data, sample size is $N = 5,000$ and $\epsilon_q i, i \geq .75$.

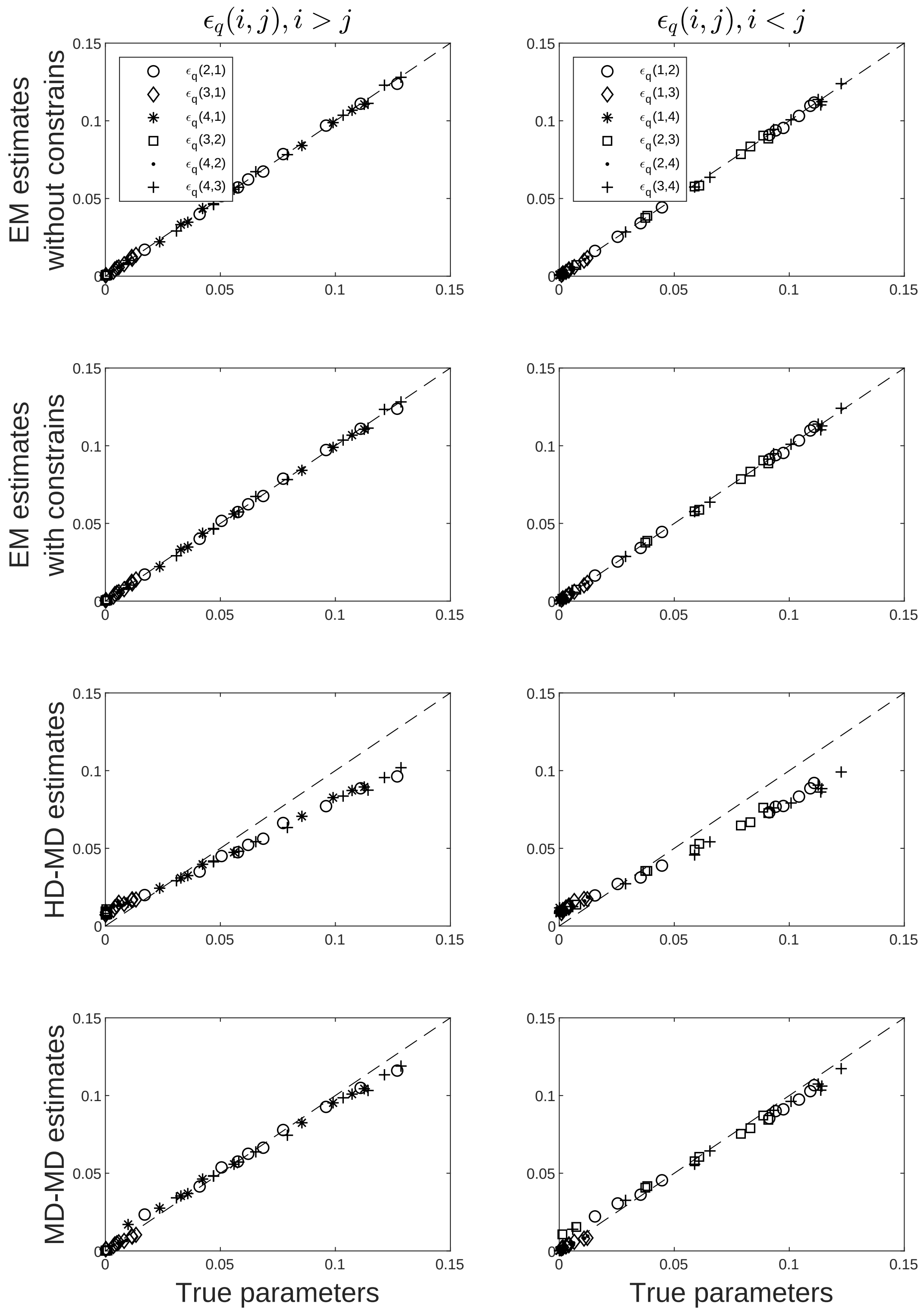

Figure 6: Parameter recovery of the PoLIM obtained by the four estimation procedures when monotonicity constraints hold in the data, sample size is $N = 2,000$ and $\epsilon_q i, i \geq$ .85.

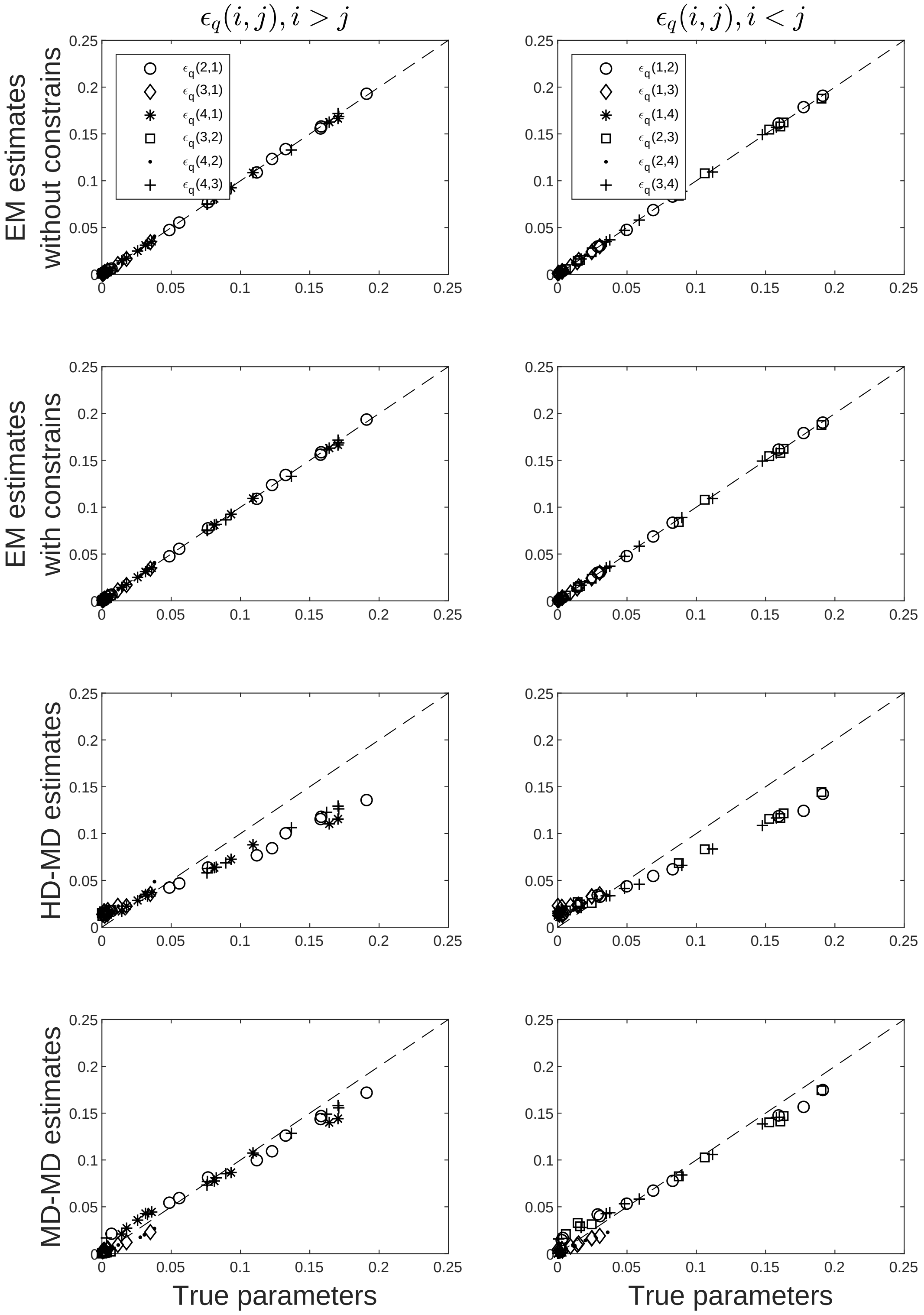

Figure 7: Parameter recovery of the PoLIM obtained by the four estimation procedures when monotonicity constraints hold in the data, sample size is $N = 2,000$ and $\epsilon_q i, i \geq .75$.

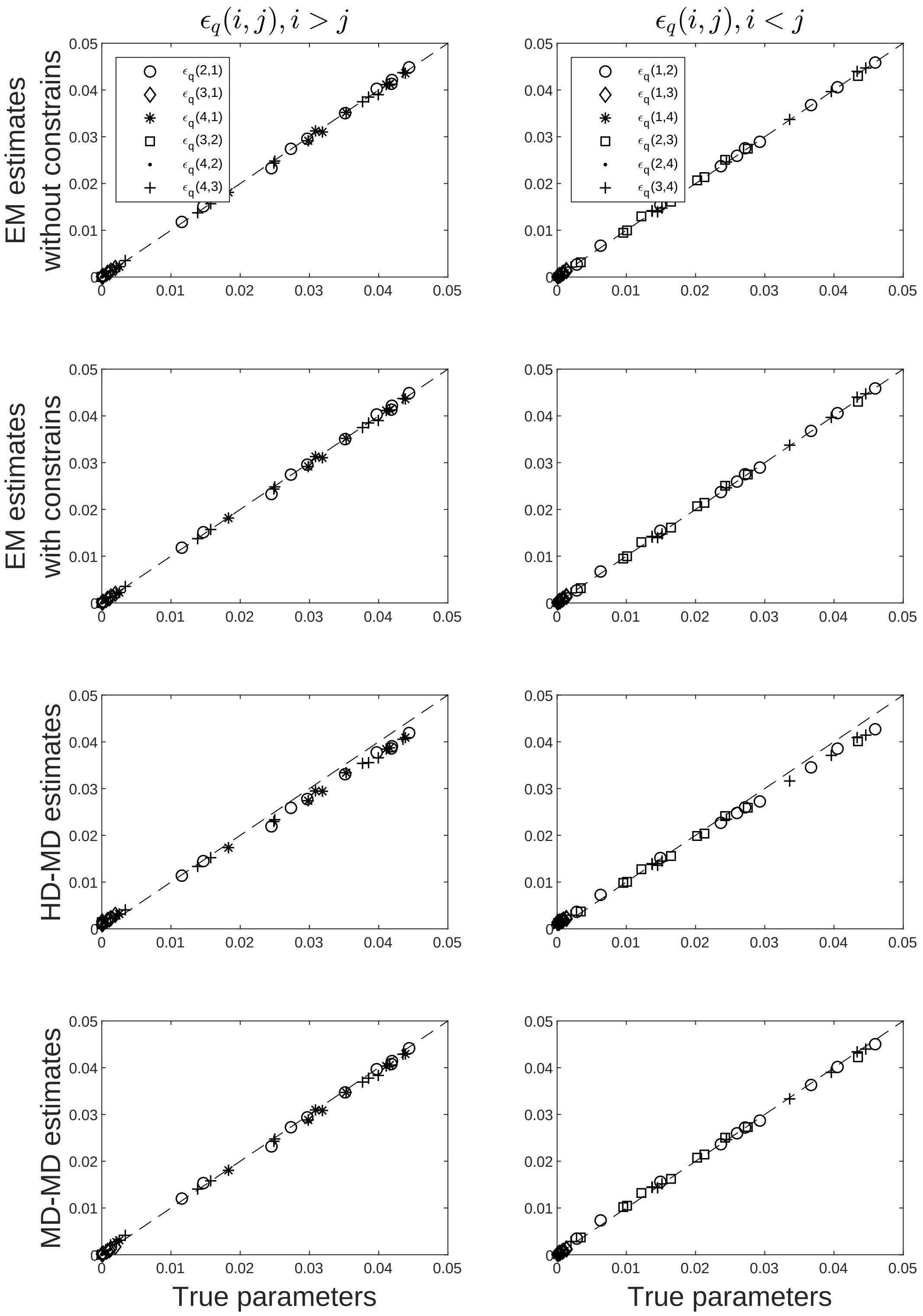

Figure 8: Parameter recovery of the PoLIM obtained by the four estimation procedures when monotonicity constraints hold in the data, sample size is $N = 5,000$ and $\epsilon_q i, i \geq .95$.

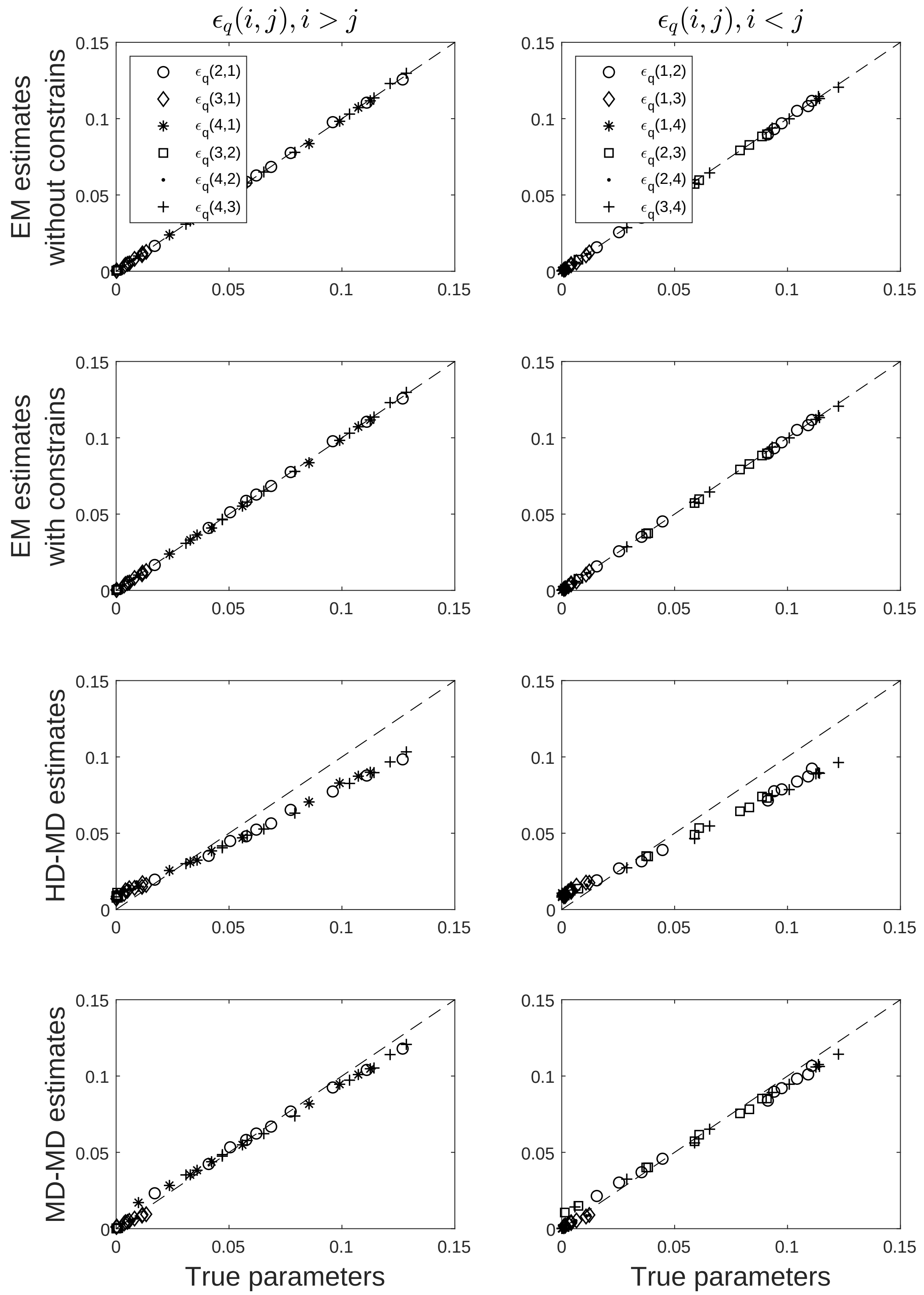

Figure 9: Parameter recovery of the PoLIM obtained by the four estimation procedures when monotonicity constraints hold in the data, sample size is $N = 5,000$ and $\epsilon_q i, i \geq .85$.

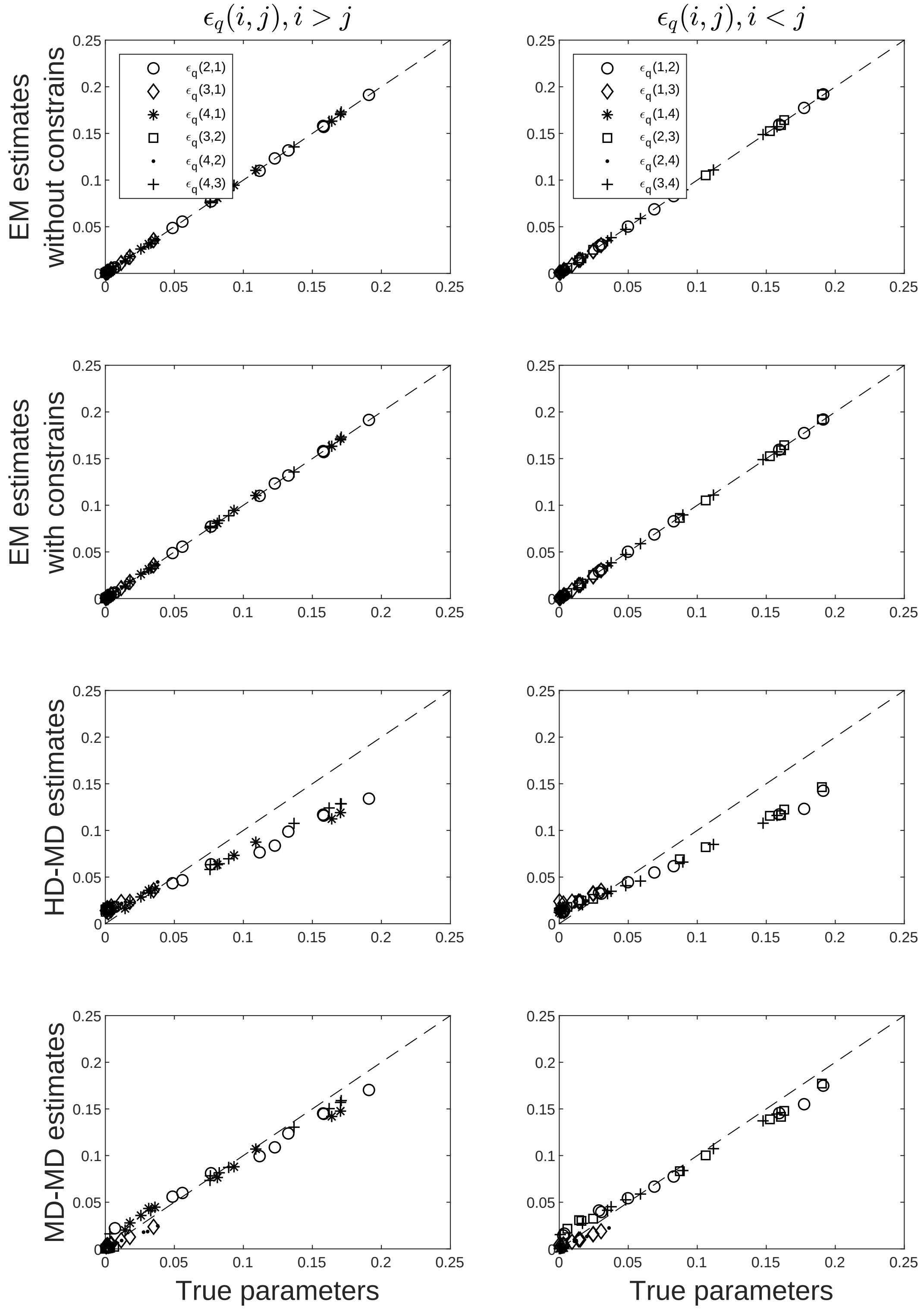

Figure 10: Parameter recovery of the PoLIM obtained by the four estimation procedures when monotonicity constraints hold in the data, sample size is $N = 5,000$ and $\epsilon_q i, i \geq .75$.

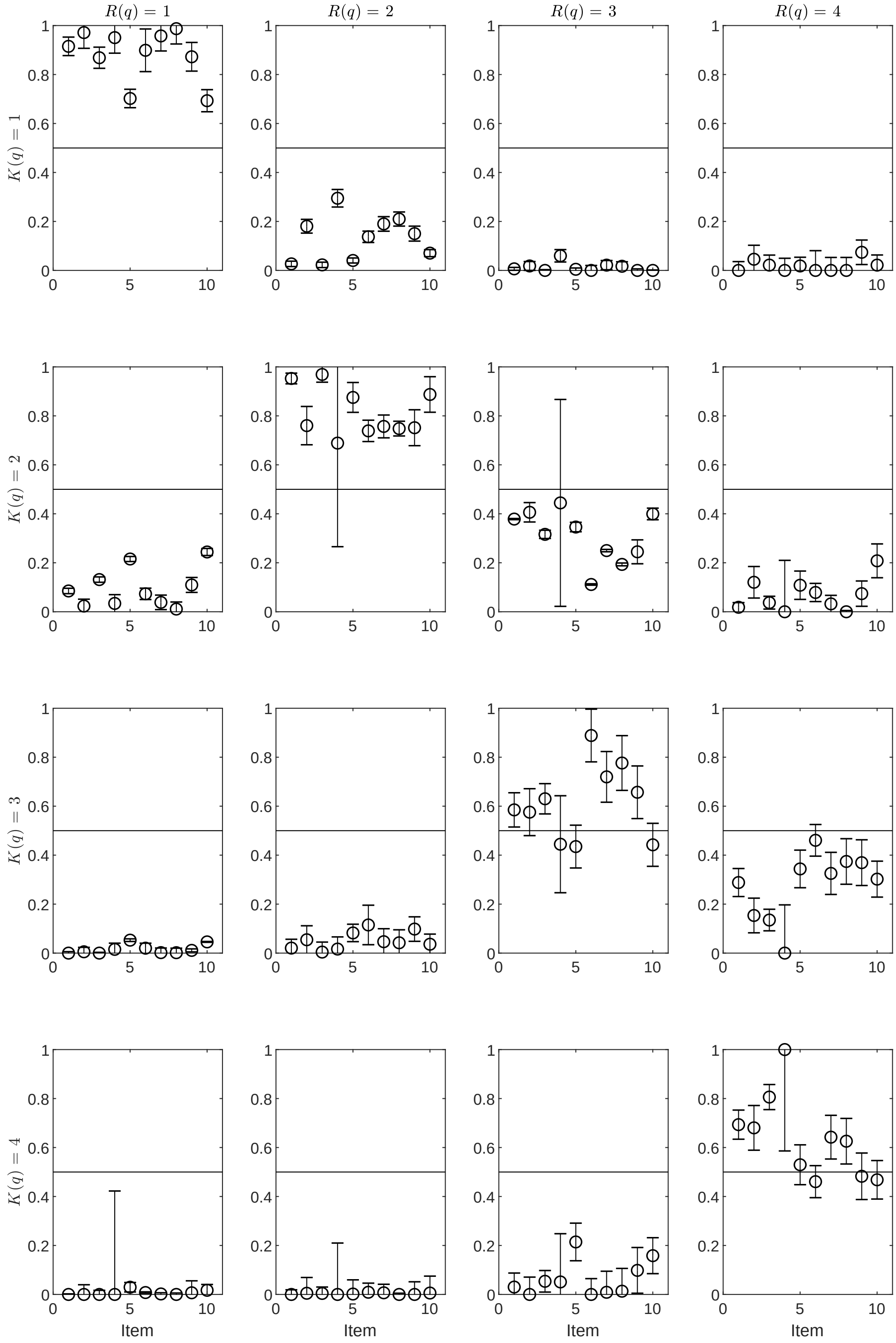

Figure 11: Parameter estimates of the constrained PoLIM obtained in the empirical application.
